# The Impact of Nylon-3 Copolymer Composition on the Efficiency of siRNA Delivery to Glioblastoma Cells

**DOI:** 10.3390/nano9070986

**Published:** 2019-07-08

**Authors:** Natascha Hartl, Friederike Adams, Gabriella Costabile, Lorenz Isert, Markus Döblinger, Ximian Xiao, Runhui Liu, Olivia M. Merkel

**Affiliations:** 1 Pharmaceutical Technology and Biopharmaceutics, Ludwig-Maximilians-University, Butenandtstr. 5-13, 81377 Munich, Germany; 2 Functional Nanosystems, Department Chemistry and Biochemistry, Ludwig-Maximilians-University, Butenandtstr. 11, 81377 Munich, Germany; 3 State Key Laboratory of Bioreactor Engineering, Key Laboratory for Ultrafine Materials of Ministry of Education, Research Center for Biomedical Materials of Ministry of Education, East China University of Science and Technology, Shanghai 200237, China

**Keywords:** nylon-3 polymers, siRNA delivery, polyplexes, hydrophobically modified cationic polymers, glioblastoma

## Abstract

Glioblastoma multiforme is a devastating disease that has attracted enormous attention due to poor prognosis and high recurrence. Small interfering RNA (siRNA) in principle offers a promising therapeutic approach by the downregulation of disease-related genes via RNA interference. For efficient siRNA delivery to target sites, cationic polymers are often used in preclinical studies for the protection of siRNA and complex formation based on electrostatic interactions. In an effort to develop biocompatible and efficient nanocarriers with a translational outlook for optimal gene silencing at reduced toxicity, we synthesized two sets of nylon-3 copolymers with variable cationic content (DM or NM monomer) and hydrophobic subunits (CP monomer) and evaluated their suitability for in vitro siRNA delivery into glioblastoma cells. DM_0.4_/CP_0.6_ and NM_0.4_/CP_0.6_ polymers with similar subunit ratios were synthesized to compare the effect of different cationic subunits. Additionally, we utilized NM_0.2_/CP_0.8_ polymers to evaluate the impact of the different hydrophobic content in the polymer chain. The siRNA condensation ability and polymer–siRNA complex stability was evaluated by unmodified and modified SYBR gold assays, respectively. Further physicochemical characteristics, e.g., particle size and surface charge, were evaluated by dynamic light scattering and laser Doppler anemometry, whereas a relatively new method for polyplex size distribution analysis—tunable resistive pulse sensing—was additionally developed and compared to DLS measurements. Transfection efficiencies, the route of cell internalization, and protein knockdown abilities in glioblastoma cells were investigated by flow cytometry. Furthermore, cellular tolerability was evaluated by MTT and LDH assays. All the polymers efficiently condensed siRNA at N/P ratios of three, whereas polymers with NM cationic subunits demonstrated smaller particle size and lower polyplex stability. Furthermore, NM_0.2_/CP_0.8_ polyplexes with the highest hydrophobic content displayed significantly higher cellular internalization in comparison to more cationic formulations and successful knockdown capabilities. Detailed investigations of the cellular uptake route demonstrated that these polyplexes mainly follow clathrin-mediated endocytotic uptake mechanisms, implying high interaction capacity with cellular membranes. Taken together with conducive toxicity profiles, highly hydrophobic nylon-3 polymers provide an appropriate siRNA delivery agent for the potential treatment of glioblastoma.

## 1. Introduction

Glioblastoma multiforme (GBM) is the most common devastating type of primary malignant tumor of the central nervous system. The current standard treatment includes surgical resection followed by radiation and chemotherapy with temozolomide and the use of the monoclonal antibody bevacizumab, which inhibits vascular endothelial growth factors [1]. However, even with aggressive treatment, patient outcomes remain poor with median survival times of only 12 to 15 months [2]. Thus, any new therapeutic strategy to target this disease is of significant benefit. Small interference RNA (siRNA) is a promising therapeutic approach due to its ability to potentially knock down disease-related genes, and it has been intensively investigated for the treatment of a broad range of disorders [3,4,5,6,7]. In the case of glioblastoma treatment, it was recently shown by various groups that siRNA can successfully induce glioma-related gene knockdown and tumor growth inhibition within in vitro as well as in vivo experiments [8,9,10]. Successful siRNA delivery and the silencing of glioma-related genes, e.g., B-cell lymphoma/leukemia-2 (Bcl-2), was enabled by dendrimer—or polyethylenimine (PEI)—entrapped gold nanoparticles by the groups of Qiu and Shi, respectively [11,12]. In these treatments and also in most other cases of siRNA delivery, limitations in the application of naked siRNA caused by rapid degradation, immune response, and low passive cell uptake [13] are bypassed by using suitable delivery systems to encapsulate the nucleic acids by electrostatic interactions in order to shield them from the environment and assist cellular internalization. Several non-viral siRNA formulation approaches such as liposomes and especially cationic polymeric delivery systems have been extensively investigated [14]. However, the main hurdle of high cellular toxicity due to high positive charge density and the poor transfection efficiencies of cationic nucleic acid carriers still remains [15]. As an advancement, hydrophobically modified cationic polymers, e.g., phospholipid-modified PEI [16], polyamidoamine dendrimers (PAMPAM) [17], and PEI-poly(caprolactone)-poly(ethylene glycol) (PEI-PCL-PEG) polymers [18] were investigated by various groups due to their improved performance as siRNA carriers coupled with considerably decreased cytotoxicity [19]. In addition, nylon-3 polymers, which have a similar backbone to biocompatible and biodegradable peptides, were tested for various medical/biological applications by Gellman and Liu et al. Due to their high altering amenability, they were successfully used for mimicking antimicrobial host-defense peptides [20], lung surfactant [21], natural polysaccharides [22], and as cell adhesion promoters [23], which also laid promising foundations for application as a potential gene delivery agent. The first siRNA delivery performance study of these polymers was investigated in non-small cell lung cancer cells (H1299), indicating that hydrophobic structures that are made from a considerable amount of hydrophobic subunits might be superior siRNA delivery agents [24]. Nylon-3 polymers can be synthesized via anionic ring-opening polymerization (ROP) via the statistical copolymerization of various β-lactams using lithium bis(trimethylsilylamide) (LiHMDS) as the initiator to incorporate both cationic and lipophilic/hydrophobic subunits, providing the opportunity to obtain versatile and tailor-made polymer compositions by regulation of the monomer feed [25]. Herein, we synthesized a tailored set of random nylon-3 copolymers via ROP with an increased amount of hydrophobic subunits derived from β-lactam cyclopentyl (CP) and cationic subunits either from β-lactam with dimethyl (DM) or no methyl (NM) substitution. Differently designed nylon-3 polymers concerning the ratio between the hydrophobic and cationic subunit as well as the use of different cationic monomers were used to investigate the suitability for siRNA delivery into glioblastoma cells depending on the polymer’s microstructure. Optimized NM monomers were used to decrease steric demand and the hydrophobicity of cationic subunits in order to enable more compact siRNA complexation, potentially leading to more favorable particle sizes. The obtained siRNA–polymer complexes (polyplexes) were characterized in detail regarding physicochemical characteristics such as siRNA encapsulation ability, polyplex stability, particle size, and surface charge. Tunable resistive pulse sensing (TRPS) was established as a suitable method for polyplex size distribution analysis by comparison to dynamic light scattering (DLS) data. Furthermore, cellular internalization, the route of uptake, and gene knockdown efficiency were assessed by flow cytometry, and cell tolerability was examined by MTT and LDH assays. Based on these findings, the most hydrophobic nylon-3 polymers provide optimal properties regarding particle characteristics and the ability to fuse with cell membranes, leading to excellent transfection efficiencies and successful gene knockdown in glioblastoma cells with minimal cytotoxic effects.

## 2. Materials and Methods

### 2.1. Materials

HEPES (4-(2-hydroxyethyl)–1-piperazineethanesulfonic acid), sodium acetate, potassium chloride, Tween^®^ 20, heparin sodium salt, thiazolyl blue tetrazolium bromide (MTT), nystatin, wortmannin, chlorpromazine hydrochloride, methyl-β-cyclodextrin, chloroquine diphosphate, paraformaldehyde solution, 4′,6–diamidino–2-phenylindole dihydrochloride (DAPI), FluorSave Reagent, and for cell culture U87 cells (human glioblastoma astrocytoma), Eagle’s Minimum Essential Medium (EMEM), RPMI-1640 Medium, fetal bovine serum (FBS), Penicillin-Streptomycin solution, Dulbecco’s Phosphate Buffered Saline (PBS), trypsin-EDTA solution 0.25%, 200 mM of L-glutamine solution, dimethyl sulfoxide (DMSO), and Geniticin (G418) disulfate solution were purchased from Sigma-Aldrich (Taufkirchen, Germany). Green fluorescent protein (eGFP) reporter cell line–NCI-H1299 (human non-small cell lung carcinoma) was purchased from ATCC (Manassas, VA, USA). SYBR Gold Dye, Lipofectamin 2000 transfection reagent, AlexaFluor 488 (AF488), and 647 (AF647) dyes were purchased from Life Technologies (Carlsbad, CA, USA). HyClone trypan blue solution 0.4% in phosphate-buffered saline was obtained from FisherScientific (Hampton, NH, USA) and CytoTox 96^®^ Non-Radioactive Cytotoxicity Assay was purchased from Promega (Madison, WI, USA). Amine-modified eGFP siRNA (5′-pACCCUGAAGUUCAUCUGCACCACcg, 3′-ACUGGGACUUCAAGUAGACGGGUGGC), human glyceraldehyde 3–phosphate dehydrogenase (GAPDH) siRNA (5′-pGGUCGGAGUCAACGGAUUUGGUCgt, 3′-UUCCAGCCUCAGUUGCCUAAA-CCAGCA), and scrambled siRNA (5′-pCGUUAAUCGCGUAUAAUACGCGUat, 3′-CAGCAAUUAGCGCAUAUUAUGCGCAUAp) were purchased from Integrated DNA Technologies (Leuven, Belgium). An indication of modified nucleotides: “p” denotes a phosphate residue, lower case letters are 2′-deoxyribonucleotides, capital letters are ribonucleotides, and underlined capital letters are 2′-O-methylribonucleotides.

### 2.2. Synthesis and Characterization of Nylon-3 Random Copolymers

Nylon-3 random copolymers were synthesized via the anionic ring-opening polymerization (ROP) of racemic β-lactams. Polymer DM_0.4_/CP_0.6_ (DM as cationic monomer and CP as hydrophobic monomer) was synthesized as described previously [24,25]. The polymerization was conducted in the presence of (*±*)-7-(2-tritylthioacetyl)-7-azabicyclo[4,2,0]octan-8-one (I) as the co-initiator and lithium bis(trimethylsilylamide) (LiHMDS) as the base to afford the desired polymer with one N-terminal thiol-end group per polymer chain (Figure S1, Supplementary Materials) [23]. Monomers β-NM (cationic monomer) and CP (hydrophobic monomer) for NM/CP copolymers were prepared according to literature procedures [26]. Random copolymers from β-NM and CP were synthesized by following previously reported procedures [20]. The polymerization was conducted in the presence of 4-tert-butyl-benzoyl chloride (II) as the co-initiator and LiHMDS as the base to afford the desired polymers with an N-terminal tert-butyl-benzoyl group (Figure S2, Supplementary Materials) [27]. The deprotection of all Boc-protected polymers was performed in trifluoroacetic acid (TFA) to obtain the TFA salts of the desired polymers [28]. To determine the average molecular weight and polydispersity of DM/CP polymers, characterization was conducted with Boc-protected side chains via gel permeation chromatography (GPC) using a Shimadzu GPC instrument equipped with two Waters columns (Styragel HR 4E, particle size 5 µm) linked in series, equipped with a multiangle light scattering detector (Wyatt miniDAWN, 690 nm, 30 mW) and a refractive index detector (Wyatt Optilab-rEX, 690 nm). THF was used as the mobile phase at a flow rate of 1 mL/min. The ^1^H-NMR spectra of the DM/CP polymer were measured on a Varian Mercury Nuclear Magnetic Resonance (NMR) Spectrometer at 300 MHz in deuterium oxide with 512 scans. ^1^H-NMR spectra of NM/CP polymers were measured on a Bruker AV500 in deuterium oxide with 128 scans. Molar masses of NM/CP polymers were directly calculated via ^1^H-NMR in D_2_O performed with unprotected TFA salts by comparing the signal between 4.0–4.5 ppm (one proton per repeating unit p (p = n + m)), and the tert-butyl group of the end group (1.33 ppm) or the aromatic benzylic protons (7.5–8.0 ppm).

### 2.3. Preparation of Polyplexes

To prepare polymer–siRNA complexes (polyplexes), aqueous polymer stock solutions were diluted with 10 mM of freshly filtered HEPES buffer (pH 7.2) to predetermined concentrations, which were added to a defined amount of siRNA in a microcentrifuge tube to obtain polyplexes at various N/P ratios and incubated for 30 min to permit stable polyplex formation. The N/P ratio is defined as the molar ratio between the polymer amine groups (N) and the siRNA phosphate groups (P). The amount of polymer needed to obtain different N/P ratios was calculated according to the following equation:

m (polymer in pg) = n siRNA (pmol) *×* M protonable unit (g/mol) *×* N/P *×* number of nucleotides siRNA

**Scheme 1:**
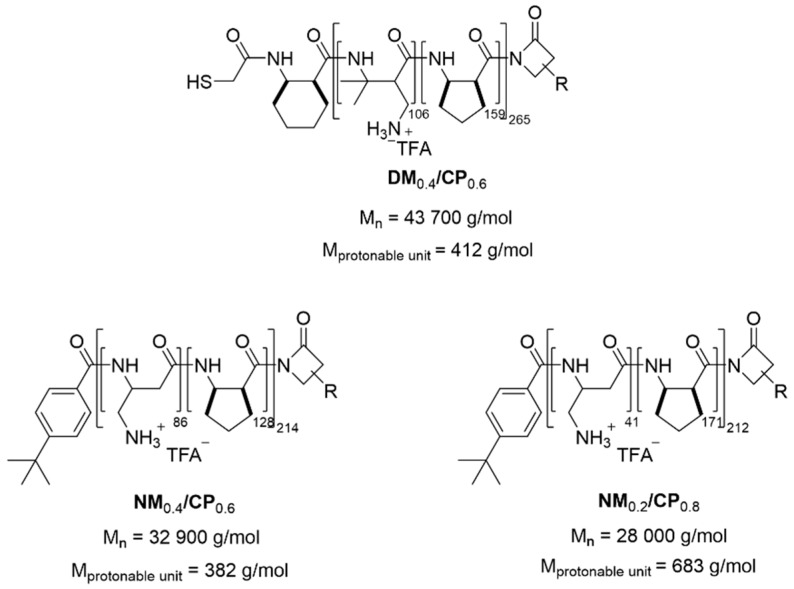
Nylon-3 polymers used in this study.

The protonable unit of each polymer was calculated by dividing its molar mass by the number of protonable primary amines present in each polymer and illustrated in Scheme 1 (number of nucleotides of 25/27mer siRNA = 52).

### 2.4. siRNA Encapsulation Assay by SYBR Gold Assay

SYBR Gold assay was used to evaluate the capacity of the polymers to condense siRNA at various N/P ratios analogous to the procedures previously described [29]. Polyplexes with 50 pmol of siRNA were prepared in HEPES buffer, and 100 µL of each polyplex solution was distributed in a white FluoroNunc 96-well plate (FisherScientific, Hampton, NH, USA). A 4X SYBR Gold solution (30 μL) was added to each well, and the plate was incubated for 10 min in the dark. The fluorescence signal was determined by using a fluorescence plate reader (FLUOstar Omega, BMG Labtech, Ortenberg, Germany) at 492 and 555 nm excitation and emission wavelengths, respectively. An analogous procedure with free siRNA was used as 100% value. Measurements were performed in triplicate, and the results are shown as mean values (*n* = 3).

### 2.5. Size and Zeta (ζ)-Potential Analysis by Dynamic Light Scattering and Laser Doppler Anemometry

The particle size, polydispersity index (PDI), and zeta potential of polyplexes were measured using a Zetasizer Nano ZS (Malvern Instruments, Malvern, UK). Polyplexes were formed at various N/P ratios in HEPES buffer. A total volume of 100 µL of each sample was added to a disposal cuvette (Malvern Instruments, Malvern, UK) and used for particle size and PDI measurements by dynamic light scattering at 173° backscatter angle running 15 scans three times per sample. Zeta potentials were measured using a Zeta Cell (Zetasizer Nano series, Malvern, UK) containing a 7X dilution of another 100-µL sample aliquot by laser Doppler anemometry (LDA) with each run consisting of 30 scans. Results are expressed as mean ± standard deviation (*n* = 3).

### 2.6. Size Measurements by Tunable Resistive Pulse Sensing

Measurements were conducted using a qNano Gold system (Izon Science, Oxford, UK) equipped with an upper and a lower fluid cell and a separating polyurethane membrane between the cells possessing a single nanopore at its center. All the measurements were performed with a NP 200 Nanopore (size range 85–500 nm) (Izon Science, Oxford, UK) with a minimum particle count of 500. The lower and upper fluid cells were filled with 75 µL and 40 µL of electrolyte (30 mM of HEPES, 100 mM of potassium chloride, 2 mM of ethylenediaminetetraacetic acid (EDTA), and 0.03% Tween^®^20), respectively. The instrument was calibrated according to the manufacturer’s protocol with defined 200-nm polystyrene calibration particles. Subsequently, the calibration particle suspension in the upper fluid cell was replaced by 40-µl polyplexes dilution. As 10 mM of HEPES buffer was not suitable for polyplex preparation to achieve a stable signal and baseline during the measurements due to insufficient electrical conductivity (σ = 44 µS/cm), samples were prepared as usual, incubated for 30 min, and 1X diluted with a freshly filtered electrolyte solution containing 30 mM of HEPES, 100 mM of potassium chloride, 2 mM of EDTA, and 0.03% Tween^®^20 (σ = 8.5 mS/cm). All the samples were used within 30 min after dilution. Membrane stretching was maintained between 44–48 mm, voltage was adjusted to achieve an appropriate baseline current for each sample, and additional pressures ranging from 6 to 18 mbar were applied to the system. The current pulse signals were collected and exported for analysis using Izon Control Suite software 3.3 (Izon Science, Oxford, UK).

### 2.7. Polyplex Morphology by Scanning Transmission Electron Microscopy Coupled with Energy-Dispersive X-ray Spectroscopy

The morphology and the composition of the polyplexes were analyzed with a Titan Themis transmission electron microscope (TEM) equipped with a Super-X energy dispersive X-ray (EDX) spectrometer. The measurements were performed in scanning transmission electron microscopy (STEM) annular dark field (ADF) mode at an acceleration voltage of 300 kV. STEM-ADF delivers atomic number sensitive contrast; thereby, areas of different composition may be distinguished from their surroundings. NM_0.2_/CP_0.8_ polyplexes were prepared in water to avoid buffer crystallization at N/P 4 and diluted 1:5 with water. A drop of particle suspension was dispensed on a plasma activated carbon-coated copper grid and left to dry for 60 s before blotting.

### 2.8. Cells and Cell Culture

U87 cells (human glioblastoma cell line) were cultured in EMEM media supplemented with heat-inactivated FBS (10%) and penicillin-streptomycin (1%). eGFP reporter cell line-NCI-H1299 was cultivated in RPMI-1640 media supplemented with heat-inactivated FBS (10%), penicillin-streptomycin (1%), and 0.4% (*v*/*v*) Geneticin (G418). The plasmid for eGFP expression also contains an antibiotic resistance for Geniticin to enable the selection of only stably eGFP expressing cells. All the cells were subcultured, maintained, and grown in an incubator in humidified air with 5% CO_2_ at 37 °C.

### 2.9. Quantification of Cellular Uptake by Flow Cytometry

Flow cytometry was used to quantify the in vitro cellular uptake of polyplexes. Amine-modified siRNA was labeled with the fluorescence dye Alexa Fluor 488 (AF488) following the manufacturer´s protocol and purified by ethanol precipitation and spin column binding, as described previously [30]. U87 cells were seeded in 24-well plates at a density of 100,000 cells per well and incubated for 24 h at 37 °C and 5% CO_2_. For all of the uptake experiments, polyplexes were prepared with 50 pmol of siRNA-AF488 at different N/P ratios, negative controls consisted of untreated and free siRNA treated cells, while positive control cells were transfected with Lipofectamine 2000 lipoplexes, which were prepared with 50 pmol of siRNA-AF488 according to the manufacturer’s protocol. After the transfection of cells for 24 h, the incubation medium was removed, and the cells were washed with PBS and detached using 0.25% trypsin-EDTA. Samples were washed two times with PBS and resuspended in 500 µL of PBS/2 mM of EDTA. Additionally, trypan blue quenching was used to exclude the surface fluorescence signals of not completely internalized siRNA complexes. Results were compared to those obtained with cells that did not undergo trypan blue quenching. Median fluorescence intensities (MFI) were analyzed using an Attune NxT Acoustic Focusing Cytometer (Thermo Fisher Scientific, Waltham, MA, USA) by exciting the siRNA-AF488 at 488 nm and measuring the fluorescence signal with a 530/30-nm emission filter. Samples were run in triplicate, and each sample consisted of a minimum of 10,000 viable cells. Results are given as mean ± standard deviation (*n* = 3).

### 2.10. Route of cellular Uptake

To investigate the route of polyplexes uptake, experiments with different types of specific uptake inhibitors were performed [31]. U87 cells (100,000 per well) seeded 24 h prior to the experiment were incubated with nystatin (10 µg/mL), wortmannin (12 ng/mL), chlorpromazine (10 µg/mL), and methyl-beta-cyclodextrin (3 mg/mL) for 1 h followed by incubation with polyplexes containing AF488-labeled siRNA for 24 h. Positive control cells without inhibitor treatment were transfected with polyplexes, and the untreated cells served as a blank control. After trypsinizing, 20 µL of cell suspension was stained with 0.4% trypan blue solution to investigate the cytotoxicity of used inhibitors. The number of living and dead cells was counted in a Neubauer chamber using an Axio Vert.A1 microscope (Zeiss, Oberkochen, Germany). The percentage of viable cells was calculated. Remaining samples were washed, one half was mixed with 0.4% trypan solution to quench surface fluorescence, and all the samples were subjected to flow cytometric detection of siRNA uptake, as described above. The experiments were performed in triplicate. Results are shown as a percentage of median fluorescence intensity related to not inhibited positive control samples (100%).

### 2.11. siRNA Release by Heparin Competition Assay

To evaluate the polyplex stability in the presence of competing polyanions under neutral and acidic conditions, a heparin competition assay was performed. Polyplexes were prepared in the presence of two different buffers: a 10-mM HEPES buffer (pH 7.4) and a 10-mM sodium acetate buffer (pH 4.5) to enable the comparison of polyplexes’ stability at different pH values as well as at various ionic strengths. Polyplexes sample aliquots of 60 µL were dispersed into a white FluoroNunc 96-well plate, and 10 µL of beforehand prepared heparin concentrations (0.12, 0.16, 0.21, 0.27, 0.35, 0.46, 0.59, 0.77, 1 USP units/well) were added to each well. After incubation for 30 min at room temperature, 30 µL of a 4X SYBR Gold solution was added to each well, and the plate was incubated for 10 min under light exclusion. Fluorescence measurement and the calculation of a percentage of free siRNA were performed as described in Section 2.4. To obtain more precise results, each heparin concentration was added to the respective buffer and used as a blank for related samples. Measurements were performed in triplicate, and the results are shown as mean values (*n* = 3).

### 2.12. In Vitro eGFP Knockdown by Flow Cytometry

To determine whether polyplexes can efficiently knock down protein levels in cells, silencing of the enhanced green fluorescent protein reporter gene (eGFP) was quantified by flow cytometry. H1299/eGFP cells (25,000 per well) were seeded in 24-well plates in 500 µL of medium and grown for 24 h at 37 °C in humidified atmosphere with 5% CO_2_. As positive control, Lipofectamin 2000 lipoplexes formulated with 50 pmol of siRNA against eGFP (siGFP) and as negative control, polyplexes and lipoplexes containing scrambled siRNA were included. Cells were transfected with siGFP-polyplexes and controls for 48 h with or without chloroquine treatment. Subsequently, cells were trypsinized and prepared for flow cytometry measurements as described for cellular uptake experiments. The MFIs of samples were quantified using an Attune Cytometer (Thermo Fisher Scientific, Waltham, MA, USA) with a 488-nm excitation laser and a 530/30-nm emission filter. Experiments were conducted in triplicate, each sample consisted of a minimum of 10,000 cells. Results are shown as a percentage of knockdown compared with the expression level in non-transfected cells.

### 2.13. Cytotoxicity

#### 2.13.1. MTT Assay

The cytotoxicity of free polymers and polyplexes was tested via MTT assay. Therefore, 8000 U87 cells per well were plated 24 h prior in a transparent 96-well plate (FisherScientific, Hampton, NH, USA). Free polymers were diluted in pre-warmed EMEM medium to a final concentration of 5 µg/mL and 20 µg/mL. Polyplexes were freshly prepared and 10X diluted with medium as well. After consumed medium was completely removed, 100 µL of polymer or polyplex containing medium was added to each well and incubated for 24, 48, or 72 h at 37 °C and 5% CO_2_. As a positive control, DMSO 25% in medium was used. After the respective incubation times, the medium was aspirated, and 100 µL of MTT-containing medium (0.5 mg/mL in EMEM media) was added to each well. Cells were incubated for another 3 h in the incubator. Subsequently, the cell culture medium was completely removed, and insoluble purple formazan crystals that were converted from water-soluble MTT by metabolically active mitochondria [32] was dissolved in 200 µL of isopropanol. The absorption was quantified at 570 nm and corrected with background values measured at 680 nm using a microplate reader (FLUOstar Omega, BMG Labtech, Ortenberg, Germany). The experiment was performed in triplicate, and the results are shown as mean ± standard deviation normalized to percentage of viable cells in comparison to untreated cells representing 100% viability.

#### 2.13.2. LDH Assay

Cytotoxicity, caused by membrane damage after polymer/polyplex treatment, was evaluated using a CytoTox 96^®^ Non-Radioactive Cytotoxicity Assay Kit (containing lysis buffer, Cytotox 96 reagent and stop solution) according to the manufacturer´s protocol. Briefly, U87 cells were plated 24 h prior in a 96-well plate at a density of 8000 cells per well, and were treated with polymer or polyplex solutions similar to those described for the MTT assay. Samples treated with lysis buffer were used as positive control and represent 100% lactate dehydrogenase (LDH) release; untreated cells were cultivated as blank controls. In another 96-well plate, 50-µL media aliquots were mixed with 50 µL of Cytotox 96 reagent. Plates were incubated under light protection for 30 min to allow the conversion of tetrazolium salt into a red formazan product mediated via lactate dehydrogenase (LDH) enzyme [33]. Subsequently, a 50-µL stop solution was added, and absorbance was measured at 490 nm by using a microplate reader (FLUOstar Omega, BMG Labtech, Ortenberg, Germany). The percentage of cytotoxicity was calculated by the ratio of experimental LDH release and maximum LDH release. Results are graphed as mean ± standard deviation (*n* = 3).

### 2.14. Confocal Laser Scanning Microscopy

#### 2.14.1. Endosomal Entrapment

To visualize the cellular distribution of polyplexes, 50,000 eGFP H1299 cells were seeded on 13-mm microscope cover glasses (VWR, Allison Park, PA, USA), which were placed in each well of a 24-well plate. Cells were transfected with or without chloroquine treatment for 24 h with polyplexes and lipoplexes formulated with 50 pmol of AlexaFluor 647-labeled siRNA. Subsequently, cells were washed with PBS twice, incubated with a 75-nM Lysotracker red™ dnd 99 solution for 1 h at 37 °C and 5% CO_2_, washed again with PBS, and fixed using freshly prepared 4% paraformaldehyde in PBS. After washing cells with PBS twice, their nucleus was stained with DAPI at a final concentration of 1 µg/mL. The cells were finally washed with PBS twice and mounted utilizing FluorSave reagent. Fluorescence images were acquired using a laser scanning microscope (Leica SP8 inverted, Software: LAS X, Leica microsystems GmbH, Wetzlar, Germany). Diode lasers (405 nm and 638 nm) and an argon laser (552 nm) were chosen for excitation, emission was detected in blue (410–480 nm, DAPI), red (660–785 nm, AF647), and yellow (600–700 nm, Lysotracker red) channels, respectively.

#### 2.14.2. In Vitro eGFP Knockdown

For eGFP knockdown experiments, 50,000 eGFP H1299 cells were seeded on coverslips placed in a 24-well plate and treated as described for eGFP knockdown experiments. Subsequently, cells were washed with PBS twice and fixed using 4% paraformaldehyde in PBS. After washing cells with PBS twice, their nucleus was stained with DAPI at a final concentration of 1 µg/mL. The cells were finally washed with PBS twice and mounted utilizing FluorSave reagent. Slides were imaged as described above; for excitation, a diode laser (405 nm) and an argon laser (488 nm) were used, emission was detected in blue (410–483 nm, DAPI) and green (493–778 nm, eGFP) channels, respectively.

### 2.15. Statistics

Unless otherwise stated, results are given as mean value ± standard deviation. One-way ANOVA with Bonferroni multiple comparison test and two-way ANOVA were performed in GraphPad Prism software (Graph Pad Software, La Jolla, CA, USA) to calculate p-values at 95% confidence.

## 3. Results and Discussion

### 3.1. Polymer Synthesis and Characterization

Polymers were prepared with varying ratios of hydrophobic and hydrophilic β-lactams via anionic ring-opening polymerization (ROP) as described above, by simple modulation of the monomer feed. The synthesis led to two sets of random nylon-3 copolymers that contain both a hydrophobic and a cationic subunit. The hydrophobic monomer was in every case cyclopentadienyl β-lactam (CP). The cationic monomer was either a dimethyl β-lactam (DM) or a β-lactam without methyl group (NM). DM_0.4_/CP_0.6_ and NM_0.4_/CP_0.6_ polymers with similar subunit ratios were synthesized to compare the effect of different cationic subunits. Additionally, we altered the proportion of hydrophobic subunit in the NM/CP set (NM_0.4_/CP_0.6_ and NM_0.2_/CP_0.8_) to be able to evaluate the impact of different hydrophobic fractions of the polymer chains regarding particle formation, cellular internalization, and endosomal escape ability. To determine the molecular weights of the NM/CP polymers and confirm subunit ratios, polymers were characterized by ^1^H-NMR spectroscopy, as described above. Molar masses and the degree of polymerization obtained for NM_0.4_/CP_0.6_ and NM_0.2_/CP_0.8_ polymers were 32,900 g/mol and 214 and 28,000 g/mol and 212, respectively (Figures S5 and S6, Supplementary Materials). Due to the lack of visible end groups in the ^1^H-NMR spectra (Figure S4, Supplementary Materials), in order to determine the polydispersity and confirm the monomodal distribution of nylon-3 polymers, DM_0.4_/CP_0.6_ was characterized by gel permeation chromatography (GPC) in THF using the boc-protected polymer. Values derived from GPC analysis of DM_0.4_/CP_0.6_ are shown in Figure S3 (Supplementary Material). Based on the GPC results, a degree of polymerization of 265 and a molar mass of 43,700 g/mol for unprotected DM_0.4_/CP_0.6_ was calculated.

### 3.2. siRNA Encapsulation Assay

**Figure 1:**
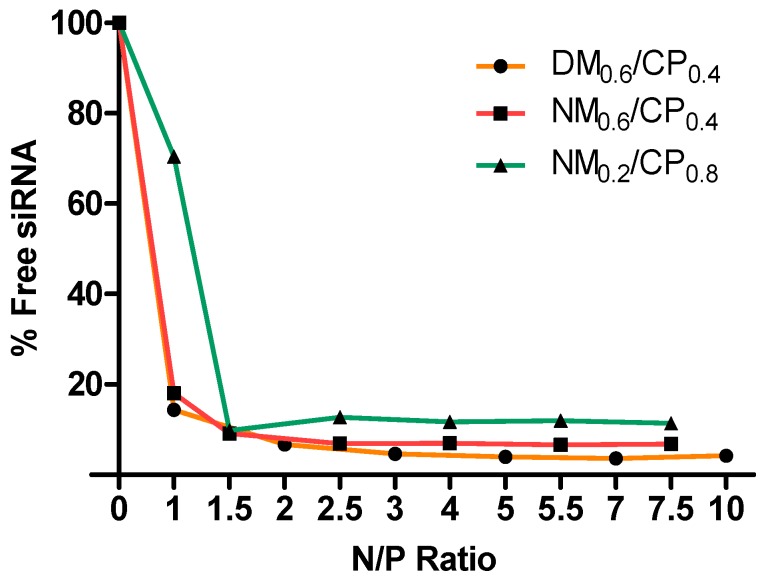
Small interfering RNA (siRNA) encapsulation profiles of polyplexes as measured by SYBR Gold assay at various N/P ratios. 100% values (N/P = 0) are represented by the determined fluorescence of uncondensed siRNA (data points indicate mean, *n* = 3). N/P ratio is defined as the molar ratio between the polymer amine groups (N) and the siRNA phosphate groups (P).

In order to enable the delivery of the siRNA molecule to target sites and especially to protect the sensitive backbone from various sources of degradation after application such as nucleases [34], an effective method of protection is encapsulation by charge complexation. Positively charged polymers electrostatically interact with negative charges provided by the phosphate groups present in the siRNA molecule [35]. Consequently, the siRNA encapsulation ability of polymers represents an important property in evaluating their suitability as siRNA carriers. Although the exact mechanism for complex formation between nylon-3 polymers and siRNA is still unknown, a combination of electrostatic and hydrophobic interactions due to the structural properties of polymers is implicated [24]. As previously described, polycationic structures cause dose-dependent toxicity; therefore, optimal polymer concentrations for efficient siRNA condensation and protection need to be evaluated [15]. In order to determine the optimal amounts of polymer, we performed siRNA encapsulation assays at various N/P ratios (Figure 1) by using the fluorescent dye SYBR Gold.

In this assay, free and unbound siRNA is completely accessible to the intercalating nucleic acid dye SYBR Gold, causing a fluorescence signal enhancement; the measured fluorescence signal decreases as soon as siRNA is protected in a polyplex. DM_0.4_/CP_0.6_ and NM_0.4_/CP_0.6_ polymers showed comparable siRNA encapsulation profiles with approximately 15% free siRNA at N/P 1 and maximum condensation at N/P 3 with 4.63% and 6.8% of non-encapsulated siRNA, respectively. NM_0.2_/CP_0.8_ polymer left 70.45% of siRNA uncondensed at N/P 1 and showed the maximum protection of the siRNA payload at N/P 2.5 with 12.68% free siRNA. As the NM_0.2_/CP_0.8_ polymer has the lowest charge density, it can be concluded that a certain amount of electrostatic interaction is needed in order to encapsulate siRNA efficiently. Furthermore, the NM_0.2_/CP_0.8_ polymer was not able to condense siRNA as efficiently as the DM_0.4_/CP_0.6_ and NM_0.4_/CP_0.6_ polymers. However, all of the polymers showed highly efficient siRNA encapsulation at rather low N/P ratios in comparison to low molecular weight polyethylenimine-based polymers, for example [36]. Using the latter, the complete condensation of siRNA was achieved only at an N/P ratio of five and higher. In contrast, as described elsewhere, amphiphilic PEI-PCL-PEG polymers achieved full siRNA condensation comparable to nylon-3 polymers at an N/P ratio of two, indicating a high nucleic acid-binding affinity of amphiphilic materials that condense with siRNA electrostatically and due to hydrophobic interactions [18]. Advantageously, the use of low polymer concentrations helps avoid unwanted side or toxic effects and reduces the incurred costs of the polymer excipient.

### 3.3. Particle Characterization

#### 3.3.1. Size and Zeta (ζ)-Potential Analysis by Dynamic Light Scattering and Laser Doppler Anemometry

**Figure 2:**
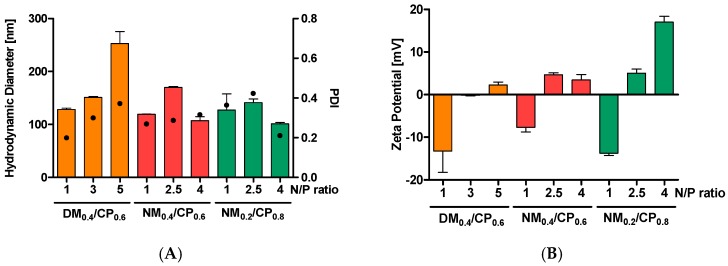
Dynamic light scattering and laser Doppler anemometry measurements of polyplexes formed with DM_0.4_/CP_0.6_, NM_0.4_/CP_0.6_, and NM_0.2_/CP_0.8_ (**A**) Hydrodynamic diameters (left y-axis), polydispersity indices (PDI, right y-axis) and (**B**) zeta potentials of DM_0.4_/CP_0.6_ polyplexes at N/P ratios of 1, 3, and 5 and of NM/CP polyplexes at N/P ratios of 1, 2.5, and 4 (data points indicate mean ± SD, *n* = 3).

In order to investigate whether nylon-3 polymer–siRNA polyplexes fulfill the general requirements for efficient nanoparticle drug delivery in glioblastoma cells [37], the first step of our study was the characterization of nanoparticles’ physicochemical characteristics, as these are two major determinants for intracellular uptake and transfection abilities. To determine optimal N/P ratios for further experiments, the hydrodynamic diameter, PDIs, and zeta potentials were measured at N/P ratios from 1 to 15 using the protocols described above, and are exemplarily shown for the NM_0.2_/CP_0.8_ polymer (Figure S7, Supplementary Material). At an N/P ratio of 4, NM_0.2_/CP_0.8_ polyplexes demonstrated the smallest particle sizes in combination with slightly positive zeta potentials, indicating optimal encapsulation efficiency at this N/P ratio. In an analogous procedure and in combination with uptake experiments (vide infra), N/P ratios for NM_0.4_/CP_0.6_ and DM_0.4_/CP_0.6_ polymers were selected. In detail, and as shown in Figure 2A, the DM_0.4/_CP_0.6_ polyplex sizes increased with increasing N/P ratios from 128.2 nm at N/P 1 to 253.0 nm at N/P 5, whereas the NM_0.4_/CP_0.6_ and NM_0.2_/CP_0.8_ polyplexes showed the smallest sizes at N/P 4 with 107.1 nm and 101.1 nm and PDIs of 0.298 and 0.193, respectively. This finding is in line with previously published data for the DM_0.4/_CP_0.6_ polymer [24]. NM/CP polymers both displayed a similar trend, as reported for high molecular weight PEI and for PEI-PCL-PEG polymers that show a minimum in hydrodynamic diameter at N/P ratios of 2 (133 nm) and 10 (128 nm), respectively. Increasing the N/P ratio beyond this optimal value was shown to cause larger hydrodynamic diameters and PDIs [38]. The zeta potentials of polyplexes increased with rising N/P ratios (Figure 2B), showing negative values at lower N/P ratios, while at N/P 4 or 5, all the polyplexes revealed positive values ranging from 2.24 mV for DM_0.4_/CP_0.6_ polyplexes to 17.03 mV for NM_0.2_/CP_0.8_ polyplexes. NM_0.4_/CP_0.6_ polyplexes showed a slightly higher positive charge at an N/P ratio of 2.5 compared to that at N/P 5, which is comparable with the findings for PEI/siRNA complexes [38].

On the basis of this data, we suggest that at an N/P ratio of one, siRNA is incompletely and loosely attached to the polymer, leading to a negative zeta potential. By increasing the N/P ratios, siRNA is more efficiently embedded in the polymers. At an N/P ratio of five for DM_0.4_/CP_0.6_ and 2.5 for both, NM/CP polymers offer sufficient polymer excess for positive surface charges. Upon the addition of further polymers, it is possible that either further polymer layers form on the surface of the polyplex or a redistribution of siRNA and polymers may occur [38]. Interestingly, NM/CP formulations demonstrated the smallest particle sizes with narrow size distributions at an N/P ratio of four, indicating higher packing efficiencies that are probably due to missing methyl groups, and therefore less steric hinderance in the cationic subunits. The NM subunit is in addition more hydrophilic, which supports interactions with siRNA molecules. These results confirmed our assumption that the NM monomer renders the polymer highly capable of forming compact particles with siRNA at favorable sizes. Increasing the N/P ratio beyond this optimal value was shown to cause larger hydrodynamic diameters and PDIs [38]. In conclusion, all the polymers at optimal N/P ratios were able to form particles with siRNA at appropriate sizes and surface charges.

#### 3.3.2. Size Measurements by Tunable Resistive Pulse Sensing

Particle diameter measurements were also performed by tunable resistive pulse sensing (TRPS). This technique has already been used in the field of drug delivery, as it provides accurate characterization possibilities of drug delivery systems to ensure effectivity and quality control [39]. The system is equipped with an upper and a lower fluid cell filled with electrolytes and separated by a polyurethane membrane containing a centered single nanopore. When a voltage is applied, ions move through the nanopore and generate a baseline current. As soon as a particle traverses the pore, a reduction in the ionic current occurs, and the magnitude of the measured blockade signal is directly proportional to the particle volume, allowing the determination of particle size. Consequently, and in contrast to DLS measurements, TRPS performs particle-by particle-measurements and provides statistical number-weighted distributions rather than average results. To allow a consistent comparison between DLS and TRPS techniques, DLS data were also expressed as number-weighted distributions calculated from scattered light intensity values and various other parameters [37]. To optimize a protocol for the measurement of polyplexes as dynamic charged systems with TRPS, different aspects had to be taken into account. As complex formation between siRNA and polymer is mainly caused by electrostatic interactions, an increase in the salt concentration, as present in measurement electrolyte, leads to a decreased binding affinity between siRNA and polymer due to charge shielding effects [40]. Furthermore, as the TRPS technique was initially optimized to measure particles with negative surface charges, we expected interactions of positively charged polyplexes with the polyurethane membrane. Modifications optimized for negatively charged particles might lead to nanopore blockades by measuring polyplexes with positive loads. In order to circumvent these limitations, we used NM_0.2_/CP_0.8_ with the lowest cationic amount for TRPS protocol optimization. By using SYBR Gold assays performed in HEPES buffer and in TRPS-electrolyte solutions, we confirmed no significant difference in encapsulation efficiencies (Figure S8A, Supplementary Material). To determine the influence on polyplex size, we performed DLS measurements in HEPES buffer and electrolyte solution over time at various N/P ratios. In line with our expectations, the hydrodynamic diameters of polyplexes increased after diluting polyplexes with electrolyte solution after 10 min of incubation due to decreased binding affinities, whereas the most distinct effect of size increase was displayed at N/P 4 (Figure S8B, Supplementary Materials). However, after 1 h of incubation time, the diameters remained quite stable. The TRPS and DLS measurements were conducted within 30 min after sample dilution to avoid the destabilizing effects of electrolytes. In Table 1, the DLS and TRPS diameters are listed for NM_0.2_/CP_0.8_ polyplexes at N/P ratios of 4, 5.5, 7.5, and 11.5.

With both techniques, the smallest particles were measured at N/P 5.5 with 133.37 nm (DLS) and 100.5 nm (TRPS). At N/P ratios of 7.5 and 11.5, diameters increased further to 144.3 nm and 164.0 nm (DLS) and to 164.00 nm and 193.00 nm (TRPS). Particle sizes depending on used N/P ratios followed the same trend, as already described above; however, due to higher ionic concentrations in the TRPS electrolyte solution, the smallest particles and consequently the most efficient siRNA packing occurred at N/P 5.5 instead of N/P 4, as determined under standard conditions in HEPES buffer. TRPS data displayed slightly higher mean diameters, but the average sizes as well as the number-weighted distribution profiles are in acceptable agreement with DLS data (Figure S9, Supplementary Materials), as estimated for monodisperse size distributions, proving successful protocol optimization. TRPS provides a promising technique for further applications in the field of polymeric drug delivery, e.g., polyplex concentration analysis, and is especially suitable for multiple population analysis, protein corona experiments, and payload release studies.

#### 3.3.3. Polyplex Morphology

**Figure 3:**
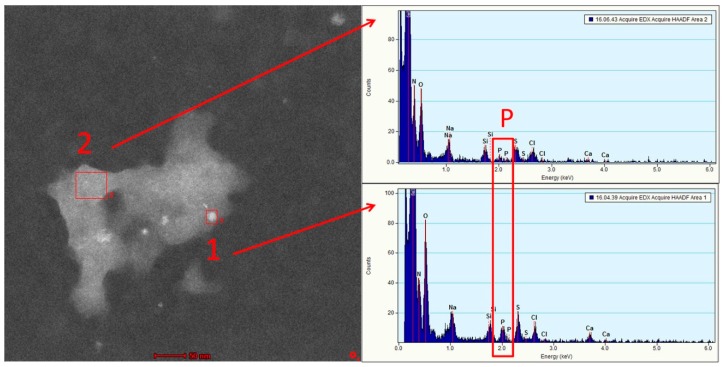
Scanning transmission electron microscopy–annular dark field (STEM-ADF) image of a typical polyplex and energy dispersive X-ray (EDX) spectra obtained from the red boxes (1) and (2). Both spectra show carbon, nitrogen, and oxygen as the main constituents. Smaller amounts of sodium, chlorine, sulfur, and calcium can be detected due to residuals of the buffer solution. Importantly, a significant amount of phosphorous can only be detected for area 1. In addition, the relative nitrogen content is considerably lower for area 1.

STEM imaging revealed non-spherical structures, as known for dynamic systems such as polyplexes [38]. The bright areas in the STEM images correspond to areas of larger electron density, which can be a matter of higher atomic number (Figure 3, area 1 (bright) and 2 (darker) in red boxes). The analysis of the compositions of brighter and darker areas by EDX elemental analysis revealed the presence of significant amounts of phosphorous and considerably lower relative nitrogen content in area 1. The area-integrated EDX spectra indicate that bright signals are enriched in phosphorous-containing siRNA and contain fewer polymers, which is characterized by a large number of amines and amides being less prominent in the bright areas.

### 3.4. Quantification of Cellular Uptake by Flow Cytometry

**Figure 4:**
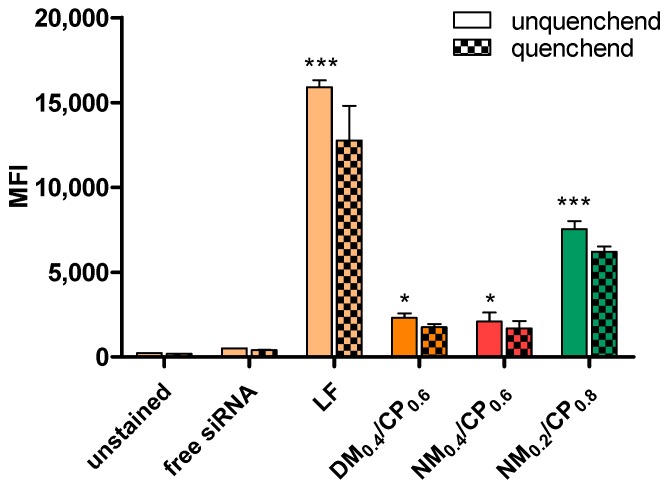
Cellular uptake of polyplexes (DM_0.4_/CP_0.6_ polyplexes: N/P 5, NM_0.4_/CP_0.6_, and NM_0.2_/CP_0.8_ polyplexes: N/P 4) after 24 h of incubation, as quantified by flow cytometry performed with and without trypan quenching and presented as median fluorescence intensity. Negative control: untreated cells and with free siRNA treated cells, positive control: with Lipofectamin (LF) lipoplexes transfected cells. (Data points indicate mean ± SD, *n* = 3, two-way ANOVA with Bonferroni post-hoc test, * *p* < 0.05, ** *p* < 0.01, *** *p* < 0.005).

As initial experiments indicated that nylon-3 polymers are suitable for siRNA delivery regarding siRNA encapsulation efficiency and physicochemical characteristics, the next step was to investigate their ability to mediate internalization into glioblastoma cells depending on their structural characteristics. In this regard, the cellular uptake of Alexa Fluor 488-labeled siRNA was quantified by flow cytometry. As cellular internalization ability can be different depending on utilized cell lines [41], a preliminary uptake experiment was conducted in H1299, in which successful transfection has been previously shown [24] and in U87 cells in order to confirm transfection abilities also in glioblastoma cells. As illustrated in Figure S10 (Supplementary Materials), the median fluorescence intensity (MFI) measured in U87 cells eventuated in overall lower MFI values, nonetheless showing the same trends regarding tested polymers and N/P ratios. This result confirmed the comparable cell entry mechanism of polyplexes in H1299 and U87 cells; however, it also indicated that U87 cells are generally harder to transfect. Based on these data, incubation times of 24 h were chosen for further experiments in U87 cells. In addition, uptake measurements at N/P ratios from 1 to 15 were conducted using the protocols described above, and are exemplarily shown for NM_0.2_/CP_0.8_ polymer (Figure S7, Supplementary Material) to verify N/P 4 as the most suitable N/P ratio for NM_0.2_/CP_0.8_. The fluorescence signals of cellular internalized siRNA-AF488 increased for N/P ratios from 4 to 7.5 and decreased for N/P ratios from 11.5 to 15. However, as significant uptake was already reached at an N/P ratio of 4, this N/P ratio was identified as optimal and used for further experiments. In an analogous procedure, N/P ratios of 4 and 5 for NM_0.4_/CP_0.6_ and DM_0.4_/CP_0.6_ polymers were determined. Figure 4 shows the MFI of U87 cells transfected with polyplexes formulated with different polymers at the preassigned N/P ratios for 24 h, in comparison to untreated cells and free siRNA treated cells as negative controls and Lipofectamin 2000 (LF) lipoplexes treated cells as positive control.

All the polyplexes displayed significantly higher uptake compared to the negative control as indicated by the statistics in Figure 4, whereby signals of cells treated with NM_0.2_/CP_0.8_ polyplexes (MFI = 7536) were approximately four times higher than those of cells treated with formulations containing higher amounts of cationic monomers (DM_0.4_/CP_0.6_ polyplexes: MFI = 2303, NM_0.4_/CP_0.6_ polyplexes: MFI = 2098). Trypan blue quenching, which was additionally performed in order to exclude extracellular fluorescent signals caused by cell surface-bound siRNA, resulted in insignificantly lower MFI values for all the tested polyplexes, indicating that inconsiderable amounts of polyplexes stuck to the outer cell membranes. The most efficient siRNA delivery in glioblastoma cells was observed by the most hydrophobic NM_0.2_/CP_0.8_ polyplexes. Besides already described interactions of cationic polyplex structures with anionic cell surface proteoglycans [42], we therefore suggest that also hydrophobic interactions with lipid bilayers might play an important role in polyplex uptake procedure. As recently described, various hydrophobically modified polymers were used to enhance the transfection efficiencies at low cytotoxicity. For example, oleic and stearic acid-modified PEI2kDa resulted in a threefold increased siRNA delivery to B16 melanoma cells in comparison to unmodified PEI [43] and triblock copolymeric systems of polyamidoamine (PAMAM) dendrimers modified with PEG and dioleoylphosphatidyl ethanolamine (DOPE) displayed significantly higher uptake of siRNA in A549 cells [44]. These results strongly support our findings where the hydrophobization of polymers enhanced siRNA delivery to target cells, even at low N/P ratios. In general, nanoparticles with a size range of 100 to 200 nm and slight positive surface charges have been shown to achieve the best cellular uptake [45]. Altogether, these results indicate that due to the accumulation of favorable properties, especially NM_0.2_/CP_0.8_ polyplexes exhibit great potential as siRNA delivery agents.

### 3.5. Route of Cellular Uptake

**Figure 5:**
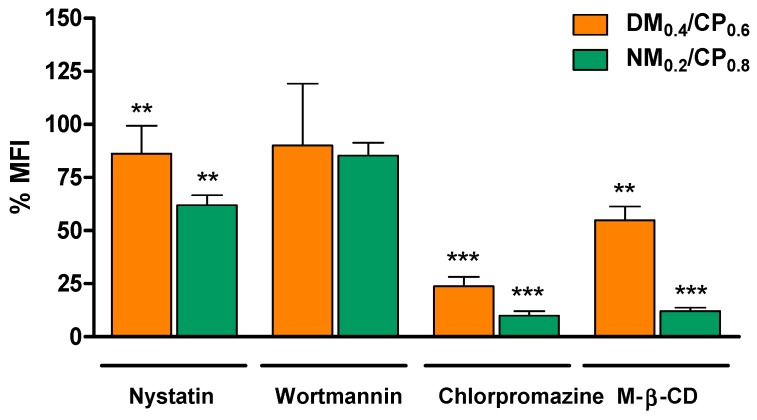
Cellular uptake of polyplexes (DM_0.4_/CP_0.6_ polyplexes: N/P ratio = 5 and NM_0.2_/CP_0.8_ polyplexes: N/P ratio = 4) after treatment with nystatin (10 µg/mL), wortmannin (12 ng/mL), chlorpromazine (10 µg/mL), and methyl-β-cyclodextrin (M-β-CD) (3 mg/mL) as evaluated by flow cytometry and presented as median fluorescence intensities (MFI). (Results are shown as mean ± SD as percentage of median fluorescence intensity related to not inhibited samples, *n* = 3, two-way ANOVA with Bonferroni post-hoc test, ** *p* < 0.01, *** *p* < 0.005).

As the route of cellular uptake determines intracellular processing and the subsequent transfection efficiencies of delivery systems, it is important to profile their cellular endocytotic pathways. It was recently shown that lipoplexes are internalized solely by clathrin-mediated endocytosis, whereas polyplexes are taken up both by clathrin-mediated and caveolae-mediated endocytosis. However, it was also stated that only the caveolae-dependent route leads to successful transfection due to the lysosomal degradation of polyplexes and their payload after clathrin-mediated cell entry [41]. To investigate the route of uptake of nylon-3 polyplexes, we performed a modified cellular uptake experiment with DM_0.4_/CP_0.6_ and NM_0.2_/CP_0.8_ polyplexes. In this regard, we incubated cells with different chemical uptake inhibitors (nystatin, wortmannin, chlorpromazine, and methyl-β-cyclodextrin) prior to transfection. Subsequently, samples were treated as described above and subjected to flow cytometric measurements. Nystatin is known to inhibit the internalization of caveolae and lipid rafts through the depletion of cholesterol in the cell membrane [46]; wortmannin has been shown to block micropinocytosis, which is discussed as an alternative endocytic pathway for polyplexes. Chlorpromazine suppresses clathrin-coated pit formation by the reversible translocation of clathrin from the plasma membrane to intracellular vesicles [47]; methyl-β-cyclodextrin inhibits cholesterol-dependent clathrin-mediated glycolipids, and is involved in lipid raft depletion [48]. Several chemical inhibitors are described in the literature as substances with cell line-dependent toxic effects, leading to inaccurate experimental results. Therefore, applied concentrations need to be optimized. Herein, cell viabilities of U87 cells at chosen inhibitor concentrations were confirmed by the trypan blue staining of dead cells after inhibitor incubation (Figure S11, Supplementary Materials). As shown in Figure 5, cellular uptake after inhibitor treatment is given as a percentage of MFI related to uninhibited samples.

Wortmannin led to insignificant signal reduction, implying that macropinocytosis did not play a considerable role in polyplex internalization. Using nystatin, chlorpromazine, and methyl-β -cyclodextrin, the observed remaining signals in comparison to uninhibited polyplex uptake for DM_0.4_/CP_0.6_ were 86.17%, 23.77%, and 54.80% and for NM_0.2_/CP_0.8_ polyplexes 61.9%, 9.95%, and 12.03%, respectively. In summary, chlorpromazine and methyl-β-cyclodextrin most strongly inhibited polyplex cellular uptake for both formulations, indicating that both polyplexes were predominantly internalized via cholesterol-dependent clathrin-mediated endocytosis, and only partially by caveolae-mediated endocytosis. However, due to the stronger inhibitory effects on NM_0.2_/CP_0.8_ polyplexes, we suggest that polyplexes with higher hydrophobic content share more similarity with lipoplexes regarding their uptake route. As illustrated in Figure S11 (Supplementary Materials), the inhibition treatment was well tolerated by the treated glioblastoma cells. Taken together with the MFI values having insignificantly decreased after performing trypan blue quenching, as shown in Figure S12 (Supplementary Materials), it was concluded that the fluorescence decrease shown in Figure 5 was not a result of the cellular toxicity of the inhibitors, but that it also represented mainly internalized siRNA rather than the polyplexes that considerably accumulated on the cell surface. In line with our assumptions are recently reported data that show that 1,2-dioleyloxy-3-trimethylammonium propane (DOTAP) lipoplexes are mainly taken up by clathrin-mediated endocytosis, whereas PEI polyplexes no longer show transfection efficiency if caveolae-mediated endocytosis is blocked [41]. Furthermore, Lu et al. recently suggested a fusogenic mechanism between lipoplexes and cell membranes, as the depletion of cholesterol from the cell membrane prevented successful siRNA delivery [49]. In conclusion, clathrin-mediated endocytosis and fusogenic uptake mechanisms, as described for lipoplexes, seems to play an important role for amphiphilic polyplexes as well.

### 3.6. siRNA Release Assay

**Figure 6:**
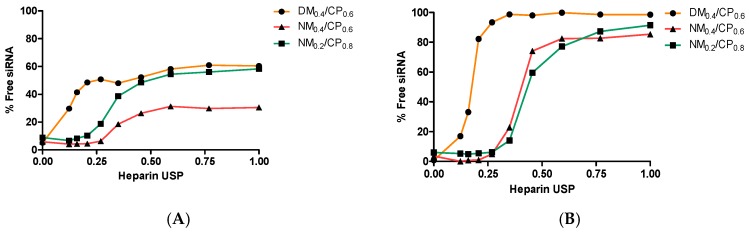
Release profiles of siRNA from DM_0.4_/CP_0.6_ polyplexes at N/P 5 and NM_0.4_/CP_0.6_ and NM_0.2_/CP_0.8_ polyplexes at N/P 4 as a function of heparin concentration (0.0–1.0 USP heparin per well) at pH (**A**) 7.4 and (**B**) 4.5 (data points indicate mean normalized fluorescence, *n* = 3).

The stability of polyplexes, which is influenced by the presence of competing anions after the addition to serum containing cell culture medium or administration in vivo, is also an important parameter to screen the potential efficiency of polymers as siRNA vectors [50]. Therefore, release assays were performed to confirm the protection ability of nylon-3 polymers for siRNA in the presence of polyanions under physiologically relevant conditions (pH 7.4). Moreover, as free siRNA that is present in the cytoplasm is a prerequisite to induce the RNAi machinery, siRNA release ability under acidic conditions (pH 4.5), mimicking the endosomal compartment, was also determined. As illustrated in Figure 6A, siRNA displacement at pH 7.4 from DM_0.4_/CP_0.6_ polyplexes was observed at low heparin concentrations and reached maximum release of approximately 60% as of 0.455 USP units heparin per well. NM_0.2_/CP_0.8_ polyplexes displayed a comparable release profile upon the addition of 0.455 USP units per well and more. NM_0.4_/CP_0.6_ polyplexes were demonstrated to be the most stable complexes, as only up to 30.5% nucleic acid was displaced when the maximum amount of heparin was added. This observation goes in line with our suggestion that missing methyl groups in NM subunits leads to both more hydrophilic character and a lack of steric hindrance, and finally to stronger interactions with siRNA molecules. The NM_0.4_/CP_0.6_ polymer contains the highest amount of NM subunits and most strongly condenses siRNA at pH 7.4. Consequently, only a small amount of siRNA molecules could be replaced by competing heparin anions. From NM_0.2_/CP_0.8_ polyplexes, siRNA was more easily replaced, which was most probably due to the lower charge density within the polymer. The methyl groups present in DM_0.4_/CP_0.6_ polymers sterically hinder complex formation with siRNA, leading to more loosely assembled polyplexes and consequently to the highest siRNA release after heparin addition. Furthermore, we assume that the effect of the dimethyl group in the DM subunit on the pKa value is negligible for the primary amines present in NM and DM subunits. We expect that the same amount of primary amines is protonated in the DM as well as in the NM subunit at pH 7.4, and therefore that obtained differences in siRNA release profiles are not influenced by the grade of protonation, but can be mainly explained by sterically hindrance mechanisms during polyplex formation.

Under acidic conditions, as illustrated in Figure 6B and in accordance with our former observations, siRNA was very easily released from DM_0.4_/CP_0.6_ polyplexes, reaching almost 100% release already at low heparin concentrations of 0.269 USP units heparin per well. NM_0.4_/CP_0.6_ and NM_0.2_/CP_0.8_ showed similar release profiles by reaching siRNA displacement of approximately 87% when using the highest heparin concentration of 1.000 USP units per well, indicating appropriate payload release abilities. SiRNA release at lower pH values might occur through charge repulsion after amine protonation leading to complex destabilization [51]. These data indicate that especially NM_0.4_/CP_0.6_ polyplexes provide a stable system in circulation, and that all the polyplexes are able to efficiently release their payload in the presence of competing anions at low pH upon being endocytosed. As described in the literature, PEI25kDa polyplexes release about 20% of siRNA at N/P 5 under physiological conditions when treated with heparin concentrations as low as 0.1 international units [36]. In comparison with nylon-3 polyplexes, they represent a system that only relies on the presence of high charge density. Under acidic conditions, PEI25kDa polyplexes were described to release up to 50% of siRNA. Due to the protonation of amines in the endosomal compartment, it was hypothesized that the high amount of positive charges within the polymer may on the one hand lead to electrostatic repulsion, complex destabilization, and endosomal escape [52]. One the other hand, it is possible that said electrostatic repulsion causes a sponge-like loose complex association, but strong charge–charge interaction in specific complex areas, which may be too strong for payload release. However, the release of siRNA is a prerequisite for its incorporation into the RNAi machinery and consequently therapeutic effects. In contrast, it was furthermore described that polymers containing hydrophobic functionalities, e.g. triazine dendrimers modified with alkyl chains, showed increased stability against heparin displacement under physiological conditions in comparison to PEI25kDa [53]. This shows very well that amphiphilic polymers interact with siRNA based on hydrophobic interactions, which are not affected by competing anions such as heparin. This is especially true for NM_0.2_/CP_0.8_ polyplexes, which show comparably high stability in particular in an acidic environment despite rather low cationic content.

### 3.7. In Vitro eGFP Knockdown

**Figure 7:**
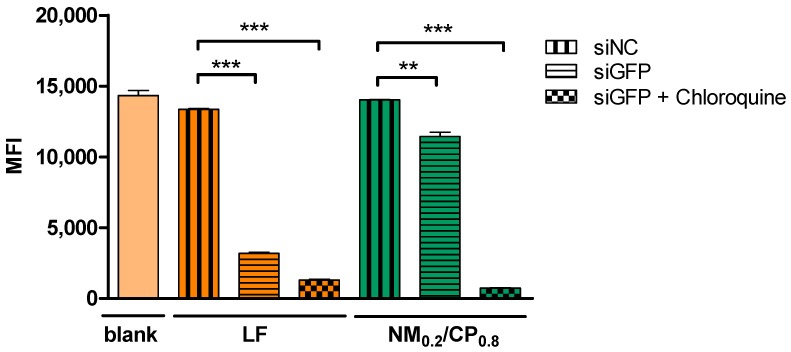
Enhanced green fluorescent protein (eGFP) knockdown of NM_0.2_/CP_0.8_ polyplexes in human non-small cell lung carcinoma cells expressing eGFP (H1299/eGFP) quantified by flow cytometry as median fluorescence intensity (MFI) of eGFP after transfection with polyplexes at N/P 4 with eGFP siRNA or scrambled control siRNA (siNC) for 48 h with and without chloroquine treatment. Blank samples consisted of H1299/eGFP untreated cells. The positive control consisted of Lipofectamin (LF) 2000 lipoplexes formulated with eGFP siRNA or scrambled control siRNA with and without chloroquine treatment (data points indicate mean ± SD, *n* = 3, two-way ANOVA with Bonferroni post-hoc test, ** *p* < 0.01, *** *p* < 0.005).

To further evaluate the gene silencing efficiency of NM_0.2_/CP_0.8_ polyplexes on the protein level, we utilized H1299/eGFP cells that stably express the ‘enhanced green fluorescent protein’ reporter gene (eGFP). H1299/eGFP cells were transfected with NM_0.2_/CP_0.8_ polyplexes formulated with siRNA against eGFP (siGFP) or with scrambled siRNA (siNC) as negative control. As positive control, Lipofectamin (LF) 2000 lipoplexes were used. Additionally, cells were treated with the endosomolytic drug chloroquine, which is able to increase the endosomal release of siRNA in order to investigate whether poor knockdown efficiencies may be caused by the endosomal entrapment of polyplexes [50]. After treatment, the median fluorescence intensity of eGFP in each sample was quantified via flow cytometry. As seen in Figure 7, lipoplexes and NM_0.2_/CP_0.8_ polyplexes achieved a significant silencing effect even without chloroquine treatment, indicating the endogenous endosomal escape capacity of at least parts of the siRNA.

However, the use of chloroquine increased the silencing effect for NM_0.2_/CP_0.8_ polyplexes tremendously, implying that endosomal entrapment of polyplexes hampers their full effect. Furthermore, NM_0.2_/CP_0.8_ polyplexes and LF lipoplexes containing scrambled control siRNA did not reduce the MFI of eGFP, indicating that the observed protein knockdown was not mediated by the polymer system or any non-specific effects, but rather was RNAi mediated by eGFP siRNA delivered into the cytoplasm. It can be suggested that parts of the siRNA were able to escape the endosome and were released into the cytoplasm. A potential endosomal membrane rupture caused by nylon-3 polyplexes might have a beneficial effect regarding endosomal escape ability. As recently described in the literature, endosome disruption can be caused by cationic as well as hydrophobic moieties of polymeric nanoparticles, which are both present in nylon-3 polymers [54]. A transbilayer flip-flop of negatively charged phospholipids from the cytoplasmatic leaflet to the luminal leaflet of the endosome was suggested to result in the formation of charge-neutral ion pairs for cationic-hydrophobic-based delivery systems. Thereby, the weakening of the electrostatic interactions between siRNA and cationic charges can cause a release of the siRNA from the complexes [54]. Furthermore, destabilization of the endosomal membrane caused by direct interactions with hydrophobic domains in the polyplexes may help release the payload into the cytoplasm [55]. Altogether, the results shown here demonstrated the knockdown ability of eGFP siRNA delivered by NM_0.2_/CP_0.8_ polyplexes, while endosomal entrapment remains the major bottleneck to achieve efficient knockdown, as described elsewhere [56]. Therefore we suggest that, in comparison to PEI, the buffering capacities of nylon-3 polymers were not sufficient to enable similar escape of the endosomal compartment, which was known for PEI formulations. This drawback can be addressed in future approaches by the precise optimization of polymer compositions. To test the nanocarriers for glioblastoma treatment in an in vivo setting in the future, it also needs to be considered that the formulation has to be able to overcome the blood–brain barrier. The ability to overcome this important barrier is currently assessed in a blood–brain barrier in vitro model.

### 3.8. Cytotoxicity

#### 3.8.1. MTT Assay

**Figure 8:**
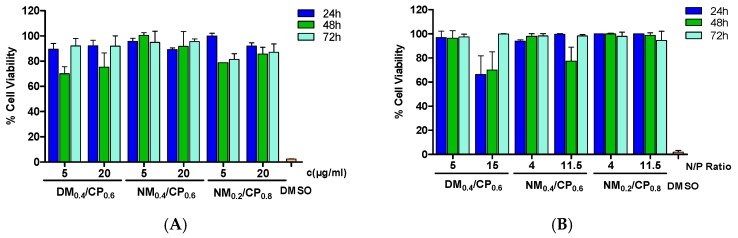
Cell viability as determined by thiazolyl blue tetrazolium bromide (MTT) assay for free polymers with concentrations of 5 µg/mL and 20 µg/mL per well (**A**) and formulated polyplexes at suitable N/P ratios (**B**) after incubation periods of 24 h, 48 h, and 72 h (results are shown as mean ± SD as percentage of viable cells in comparison to untreated cells representing 100% viability, *n* = 3).

One major drawback of cationic delivery systems is the toxicity that is caused by high positive charge densities, which leads to the cellular loss of outer membrane integrity and pore formation [15]. To test the cytotoxicity of free polymer and polyplexes, MTT assays were conducted with U87 cells that had been incubated for 24, 48, and 72 h with free polymers at two concentrations (5 µg/mL and 20 µg/mL per well) and polyplex formulations at two different N/P ratios. Thereby, lower concentration and N/P ratios represented treatment relevant conditions in in vitro experiments. The strongest toxic effect for all the polymers and polyplexes was observed after 48-h treatments, indicating that cells were able to recover after an incubation period of 72 h (Figure 8). Both concentrations that were tested for free polymers led to similar toxicity profiles. DM_0.4_/CP_0.6_ polymer demonstrated the highest negative influence on cell viability with survival rates of 70%, followed by NM_0.2_/CP_0.8_ polymer with 78.8% and NM_0.4_/CP_0.6_ polymer showing 100% viability after 48 h of incubation time (Figure 8A). In comparison to free polymers, polymer–siRNA complexes were overall better tolerated due to, as suggested, a shielding effect of positive charges after complexation with siRNA molecules (Figure 8B) [57].

At lower N/P ratios, polyplexes demonstrated no significant toxic effects. In line with the results for free polymers, DM_0.4_/CP_0.6_ polyplexes at N/P 15 caused the strongest toxic effect with approximately 70% survival rates after incubation periods of 24 h and 48 h. NM_0.4_/CP_0.6_ and NM_0.2_/CP_0.8_ polyplexes displayed survival rates of 77.4% and 98.8% after 48 h of incubation, respectively. In conclusion, acceptable cell compatibility for U87 cells could be confirmed for all free polymers as well as for all polyplexes. Free polymers with a maximum cationic content of 40% and the polyplexes formulated from them demonstrated suitable toxicity profiles in U87 cells. An already published set of nylon-3 polymers [24] showed cell viabilities ranging from 60% to 95% after 24 h of incubation at concentrations of 20 µg/mL. PEI25kDa showed significant cellular toxicity (less than 50% cell viability) even at low concentrations of 5 µg/mL of the polymer [24]. Importantly, all the nylon-3 polymers were less toxic than the broadly used high molecular weight PEI. Furthermore, this observation is especially important for future in vivo experiments, as it demonstrates that our delivery systems are well tolerated, even after potential internalization into the healthy cells of the brain tissue. Moreover, to avoid side effects in healthy cells, a promising strategy is the use of therapeutic siRNA that only target the genes that are present in tumor cells, e.g., the mutated growth factor receptor genes. In line with our assumptions, the toxicity of nylon-3 polymers could be further reduced by optimizing the content of cationic subunits within the polymer chains. However, in future designs, a critical balance should be considered between cationic charge density for siRNA complexation and adjustments that decrease polymer toxicity.

#### 3.8.2. LDH Assay

**Figure 9:**
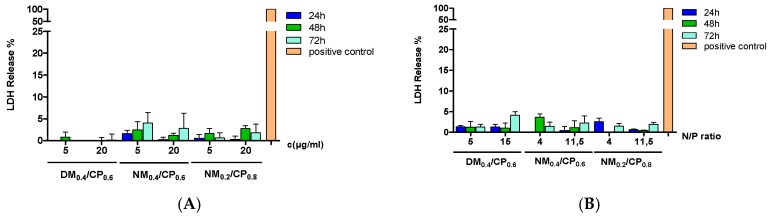
Cell membrane integrity determined by lactate dehydrogenase (LDH) assay for free polymers with concentrations of 5 µg/mL and 20 µg/mL per well (**A**) and formulated polyplexes at suitable N/P ratios (**B**) with incubation periods of 24 h, 48 h, and 72 h (results are shown as mean ± SD as percentage of LDH release in comparison to blank cells treated with lysis buffer representing 100% LDH release, *n* = 3).

We utilized lactate dehydrogenase (LDH) assays to focus additionally on the cytotoxicity caused by loss of membrane integrity. Measurements were performed with free polymers and polyplexes in U87 cells after incubation times of 24 h, 48 h, and 72 h. The percentage of LDH release for each sample was calculated in comparison to the signal of positive control cells treated with lysis buffer (100% LDH release). No considerable influence on LDH release could be found after free polymer or polyplex treatment (Figure 9).

The strongest effect on membrane integrity was demonstrated after treatment with NM_0.4_/CP_0.6_ polymers (c = 5 µg/mL, t = 72 h) and DM_0.4_/CP_0.6_ polyplexes (N/P ratio 15, t = 72 h) with 4% LDH release. Summing up, these data show that neither free polymers nor polyplexes formulated with siRNA are expected to have a noticeable effect on membrane stability. Altogether, these results demonstrated that all the formulations are well tolerated by the cellular membranes.

### 3.9. Confocal Laser Scanning Microscopy

#### 3.9.1. In Vitro eGFP Knockdown

**Figure 10:**
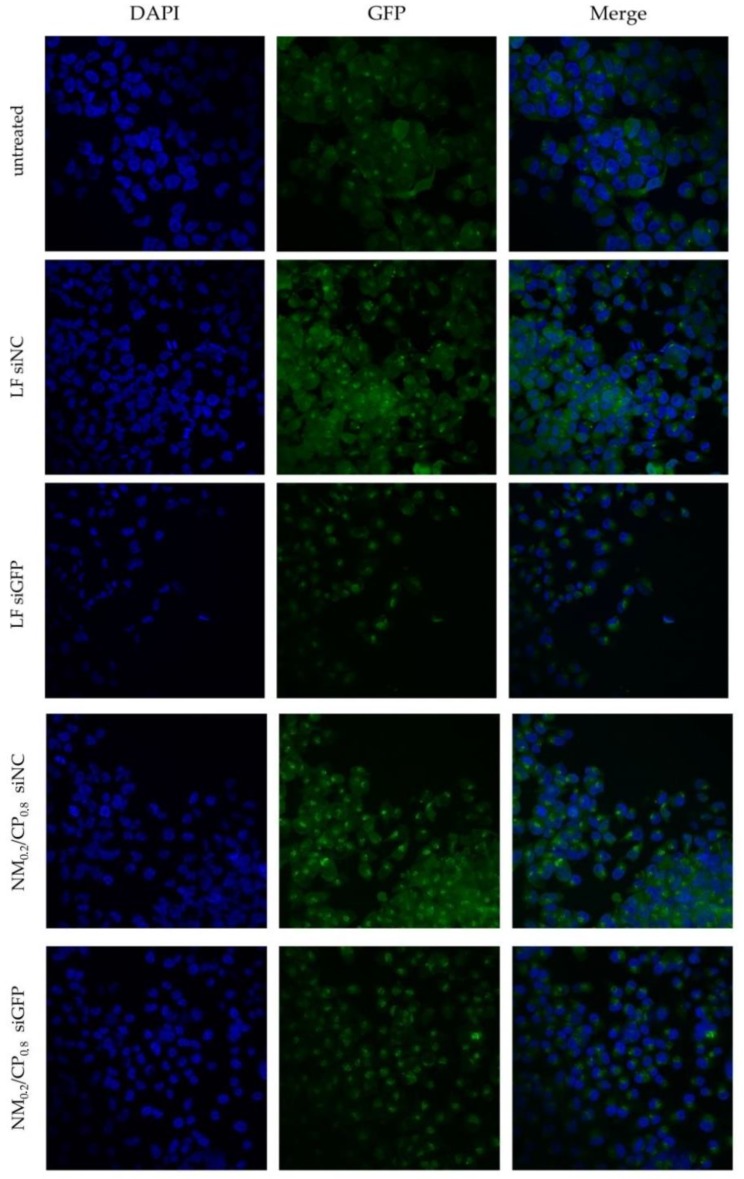
Confocal images after treatment of H1299/eGFP cells with NM_0.2_/CP_0.8_ polyplexes and Lipofectamin 2000 (LF) lipoplexes formulated with siGFP or siNC as negative control and staining with 4′,6–diamidino–2-phenylindole dihydrochloride (DAPI). eGFP fluorescence is shown in green, while the DAPI signal is shown in blue, and depicts cell nuclei.

In order to visualize the eGFP knockdown ability of NM_0.2_/CP_0.8_ polyplexes, we performed confocal microscopy experiments. H1299/eGFP cells were transfected with polyplexes formulated with siRNA against eGFP (siGFP) or with scrambled siRNA (siNC) as the negative control. As positive control, Lipofectamin (LF) 2000 lipoplexes were used. As illustrated in Figure 10, lipoplexes as well as polyplexes achieved a decrease in eGFP fluorescence in comparison to negative control samples. In line with the observations from in vitro eGFP knockdown experiments, confocal images verified partly endogenous endosomal escape capacity, even without using chloroquine as the cell organelle disruption agent.

#### 3.9.2. Endosomal Entrapment

**Figure 11:**
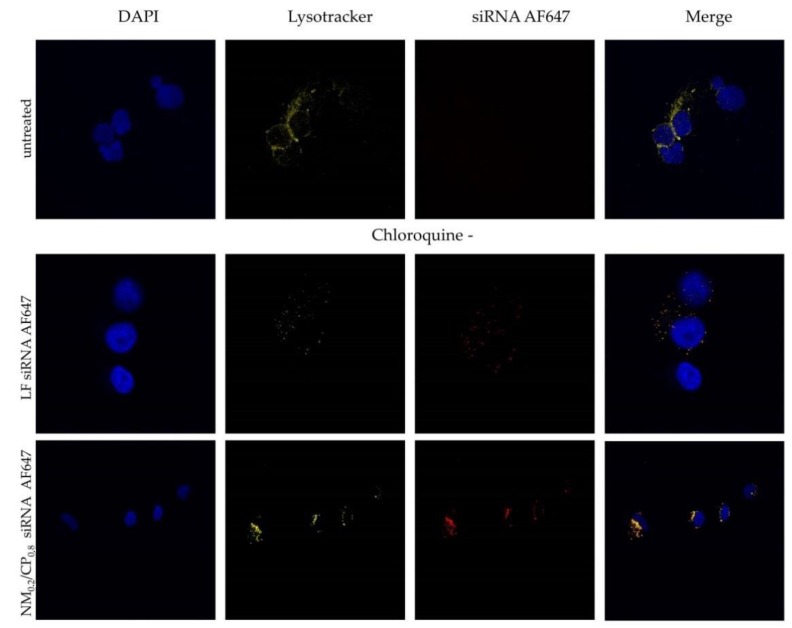

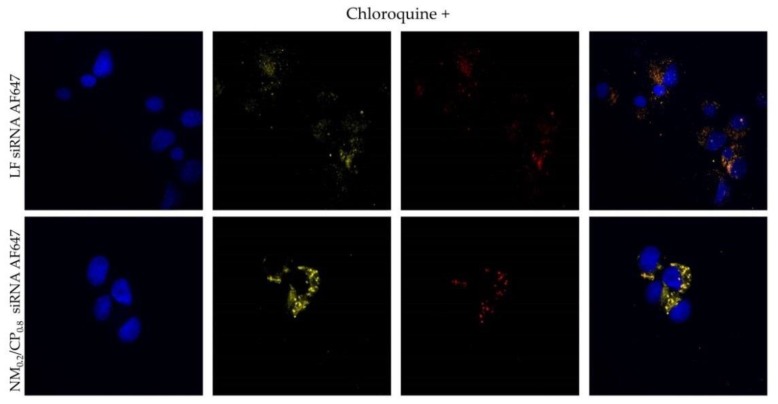
Confocal images after treatment of H1299 cells with Lipofectamin (LF) 2000 lipoplexes and NM_0.2_/CP_0.8_ polyplexes formulated with AF647-labeled siRNA (shown in red) and staining with Lysotracker red™ dnd 99 (representing lysosomes, shown in yellow) and DAPI (staining cell nuclei, shown in blue). Experiments were performed without (chloroquine–) and with chloroquine (chloroquine+) treatment.

The endosomal compartment represents a substantial barrier to successful cytosolic siRNA delivery and plays a significant role for most polyplex-based siRNA delivery systems [58]. Once polyplexes are internalized into cells via endocytotic pathways, the release of the siRNA from the endosomal compartment is a crucial prerequisite for undergoing the RNAi machinery taking place in the cytoplasm. In order to further investigate the cellular distribution of polyplexes, NM_0.2_/CP_0.8_ polyplexes were formulated with Alexa Fluor 647 labeled siRNA (shown in red). We performed a series of confocal microscopy experiments after transfection with NM_0.2_/CP_0.8_ polyplexes at N/P 4 for 24 h and the staining of acidic cell organelles such as endosomes and lysosomes with Lysotracker red™ dnd 99 (shown in yellow) and of cell nuclei with DAPI (shown in blue), utilizing Alexa Fluor 647-labeled siRNA for transfection with NM_0.2_/CP_0.8_ polyplexes at N/P 4 for 24 h (shown in red). Samples treated with endosomolytic drug chloroquine were included as positive controls. Figure 11 illustrates the resulting subcellular distribution profiles for NM_0.2_/CP_0.8_ polyplexes and Lipofectamin (LF) 2000 lipoplexes as positive control with and without chloroquine treatment. Both formulations exhibited cell internalization with rather punctuated distribution and showed colocalization with lysotracker signal indicating localization in endosomes and lysosomes. Chloroquine treatment and thus the disruption of endosomal membranes in positive control cells resulted in a more even distribution of AF647-labeled siRNA in the cytoplasm due to the facilitated endosomal escape of siRNA. These observations were consistent with our previous experiments in which NM_0.2_/CP_0.8_ polyplexes demonstrated significant cellular uptake and successful knockdown ability, but also entrapment in endosomal compartments. Future approaches may focus on polymer modification to address the bottleneck of endosomal entrapment.

## 4. Conclusions

Many cationic polymers exhibit great potential for siRNA delivery though demonstrating high cytotoxicity, relatively low transfection efficiencies, and poor biocompatibility profiles. Herein, we presented the synthesis and application of nylon-3 copolymers, consisting of hydrophobic and cationic moieties, for the encapsulation and in vitro delivery of siRNA to glioblastoma cells. Hydrophobic subunits within the polymer are derived from β-lactam monomer CP and cationic subunits either from β-lactam monomer DM or NM. DM_0.4_/CP_0.6_, NM_0.4_/CP_0.6_, and NM_0.2_/CP_0.8_ polymers were designed to study the impact of the ratio between the hydrophobic and cationic subunit as well as of the use of different cationic monomers. Efficient siRNA condensation was demonstrated for all the tested polymers, even at comparably low polymer concentrations. Regarding zeta potentials and the uptake ability of polyplexes, optimal N/P ratios of five for DM_0.4_/CP_0.6_ and four for NM/CP were investigated. The assembly of polyplexes with an optimal amount of polymers led to particles with hydrodynamic diameters of <250 nm and slightly positive surface charges, whereby the NM_0.2_/CP_0.8_ polymer formed the smallest particles at approximately 100 nm with narrow size distributions (PDI <0.2). TRPS was established as a suitable method for polyplex size distribution analysis, and provided results that were in acceptable agreement with DLS data. The method can be further investigated to enable polyplex concentration measurements in the future. In a modified SYBR Gold assay, NM_0.4_/CP_0.6_ displayed the most stable complexes under physiological conditions leading to the assumption that NM subunits interact more strongly with siRNA molecules than DM subunits. Nevertheless, all the polyplexes were able to release acceptable amounts of siRNA under acidic conditions, mimicking endosomal compartment conditions. Cellular uptake experiments, which were conducted by flow cytometry, exhibited the higher internalization abilities of NM_0.2_/CP_0.8_ polyplexes in comparison to DM_0.4_/CP_0.6_ and NM_0.4_/CP_0.6_ polyplexes. By using specific uptake inhibitors, it was confirmed that NM_0.2_/CP_0.8_ polyplexes follow endocytic uptake pathways similar to lipid nanocarriers, more precisely clathrin- and lipid-raft-mediated endocytosis. Therefore, it appears that a higher hydrophobic content has a beneficial effect on the internalization ability. Knockdown experiments showed that NM_0.2_/CP_0.8_ polyplexes were able to slightly reduce protein expression in the cytoplasm, but also that siRNA stayed predominantly entrapped in the endosome. In conclusion, the polymer composition indeed had an effect on the various crucial properties of the polyplexes, whereas NM_0.2_/CP_0.8_ with the highest hydrophobic content exhibited the most favorable characteristics. This led to excellent transfection efficiencies and successful gene knockdown in glioblastoma cells. Taken together with minimal cytotoxic effects, nylon-3 polymers were demonstrated to be a promising type of siRNA delivery agents for future approaches. Due to the simple synthesis and structure versatility of nylon-3 polymers, which have a similar backbone to biocompatible and biodegradable peptides, adjustments regarding chemical and physical properties are easily available to enhance characteristics for further improvements as siRNA delivery agents. This study confirmed that the hydrophobic modification of cationic polymers in general is a suitable tool to design drug delivery systems with enhanced cellular internalization abilities at low toxicity; however, the buffering capacities of our set of polymers were not sufficient to enable escape from the endosomal compartment. This critical point can be addressed in future approaches by the precise adjustment of polymer compositions. Ongoing work currently also focuses on including e.g., stimulus-responsive monomers in the optimized polymer to overcome the major hurdle of endosomal release.

## Figures and Tables

**Table 1:** Particle diameters of NM_0.2_/CP_0.8_ polyplexes at N/P ratios of 4, 5.5, 7.5, and 11.5 diluted 1X with electrolyte solution (30 mM of (4-(2-hydroxyethyl)–1-piperazineethanesulfonic acid) (HEPES), 100 mM of potassium chloride, 2 mM of EDTA, and 0.03% Tween^®^20) obtained by dynamic light scattering (DLS) and tunable resistive pulse sensing (TRPS) measurements (data shown as mean ± standard deviation, *n* = 3).

N/P Ratio	DLS (Mean Size ± SD) [nm]	TRPS (Mean Size ± SD) [nm]
4	158.00 ± 5.18	177.00 ± 15.56
5.5	133.37 ± 1.79	100.50 ± 0.71
7.5	144.30 ± 1.76	164.00 ± 22.63
11.5	164.00 ± 3.8	193.00 ± 5.66

## Supplementary Materials

The following results are available online at https://www.mdpi.com/2079-4991/9/7/986/s1, Figure S1. Synthesis of TFA-salt of Nylon-3 polymer DM_0.4_/CP_0.6_ in presence of a co-initiator I and a base LiHMDS. DM = dimethyl, CP = cyclopentyl, R = side chain groups of DM or CP. Adapted from [1]. Figure S2. Synthesis of TFA-salts of gene delivery Nylon-3 polymers (NM/CP) in presence of a co-initiator II and a base LiHMDS. R = side chain groups of NM or CP. Adapted from [2]. Figure S3. GPC chromatograph of Boc-protected HS-[(Boc-DM)_0.4_CP_0.6_]_265_ copolymer measured with light-scattering (red) and refractive index (blue) detectors, mobile phase: THF. Figure S4. ^1^H-NMR spectrum of unprotected DM_0.4_/CP_0.6_ polymer measured in D_2_O (300 MHz, 512 scans). Figure S5. ^1^H-NMR spectrum in D_2_O of unprotected NM_0.4_/CP_0.6_ polymer measured in D_2_O (500 MHz, 126 scans). Figure S6. ^1^H-NMR spectrum of unprotected NM_0.2_/CP_0.8_ polymer measured in D_2_O (500 MHz, 126 scans). Figure S7. (**A**) Hydrodynamic diameters investigated by DLS (left y-axis) and polydispersity indices (PDI, right y-axis), (**B**) zeta potentials measured by LDA and (**C**) MFIs of NM_0.2_/CP_0.8_ cells treated with respective polyplexes determined by flow cytometry. (Data points indicate mean ± SD, *n* = 3). Figure S8. (**A**) siRNA encapsulation profiles of NM_0.2_/CP_0.8_ polyplexes prepared in 10 mM HEPES buffer at N/P 4 and 1X diluted with either 10 mM HEPES buffer or TRPS electrolyte solution (30 mM HEPES, 100 mM potassium chloride, 2 mM EDTA and 0.03% Tween^®^20). 100% values (N/P = 0) are determined by fluorescence of uncondensed siRNA. (Data points indicate mean, *n* = 3). (**B**) DLS measurements of NM_0.2_/CP_0.8_ polyplexes prepared in 10 mM HEPES buffer at N/P 4 and 1X diluted with either 10 mM HEPES buffer or TRPS electrolyte solution, measured after 10 and 60 min incubation period (Data points indicate mean ± SD, *n* = 3). Figure S9. Number-weighted size distributions of NM_0.2_/CP_0.8_ polyplexes at N/P ratios 5, 5.5, 7.5 and 11.5. 1:1 diluted with electrolyte solution and measured by DLS and TRPS, respectively. Figure S10. Cellular uptake of polyplexes performed at various N/P ratios in (**A**) H1299 cells and (**B**) U87 cells after 5 h incubation as determined by flow cytometry presented as median fluorescence intensity. Negative control: untreated cells and cells treated with free siRNA, positive control: cells transfected with Lipofectamin (LF) lipoplexes. (Data points indicate mean ± SD, *n* = 3, two-way ANOVA with Bonferroni post-hoc test, *** *p* < 0.005). Figure S11. U87 cell viabilities after treatment with nystatin (10 µg/mL), wortmannin (12ng/mL), chlorpromazine (10 µg/mL) and methyl-beta-cyclodextrin (3 mg/mL); determined by trypan blue staining. Number of living and dead cells was counted in a Neubauer chamber using an Axio Vert.A1 microscope. The percentage of viable cells was calculated. (Results are given as mean ± SD, *n* = 3). Figure S12. Cellular uptake of polyplexes (DM_0.4_/CP_0.6_ polyplexes: N/P ratio= 5 and NM_0.2_/CP_0.8_ polyplexes: N/P ratio = 4) after treatment with nystatin (10 µg/mL), wortmannin (12 ng/mL), chlorpromazine (10 µg/mL) and methyl-β-cyclodextrin (M-ββ-CD) (3 mg/mL) conducted with and without trypan quenching as evaluated by flow cytometry and presented as MFI. (Results are shown as mean ± SD as percentage of median fluorescence intensity related to not inhibited samples, *n* = 3).
