# Supplementary material for: The Impact of Nylon-3 Copolymer Composition on the Efficiency of siRNA Delivery to Glioblastoma Cells
Source: Nanomaterials (Basel). 2019 Jul 8;9(7):986. doi: 10.3390/nano9070986 (PMC6669510; doi:10.3390/nano9070986)
Supplement: Supplementary file 1 [file nanomaterials-09-00986-s001.pdf]

nanomaterials

MDPI

# Supplementary Materials

# The Impact of Nylon-3 Copolymer Composition on the Efficiency of siRNA Delivery to Glioblastoma Cells

**Natascha Hartl [1], Friederike Adams [1], Gabriella Costabile [1], Lorenz Isert [1], Markus Döblinger [2], Ximian Xiao [3], Runhui Liu [3] and Olivia M. Merkel [1,*]**

[1] Pharmaceutical Technology and Biopharmaceutics, Ludwig-Maximilians-University, Butenandtstr. 5-13, 81377 Munich, Germany
[2] Functional Nanosystems, Department Chemistry and Biochemistry, Ludwig-Maximilians-University, Butenandtstr. 11, 81377 Munich, Germany
[3] State Key Laboratory of Bioreactor Engineering, Key Laboratory for Ultrafine Materials of Ministry of Education, Research Center for Biomedical Materials of Ministry of Education, East China University of Science and Technology, Shanghai 200237, China
* Correspondence: Olivia.merkel@lmu.de; Tel.: +49-89-2180-77025

## 1. Polymer synthesis and characterization

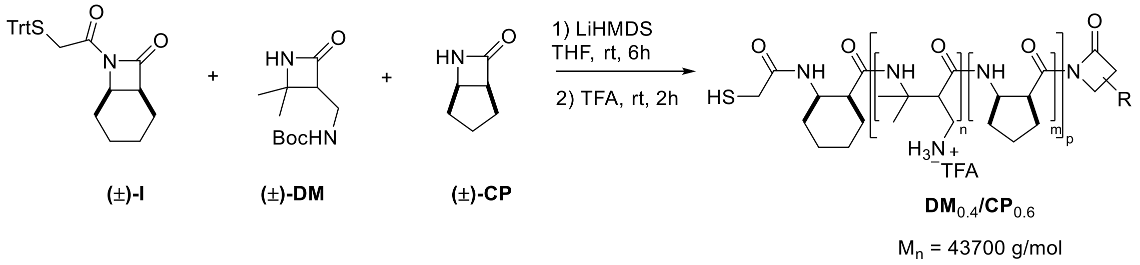


**Figure S1.** Synthesis of TFA-salt of Nylon-3 polymer $DM_{0.4}/CP_{0.6}$ in presence of a co-initiator I and a base LiHMDS. DM = dimethyl, CP = cyclopentyl, R = side chain groups of DM or CP. Reproduced with permission from [1]. Copyright American Chemical Society, 2014

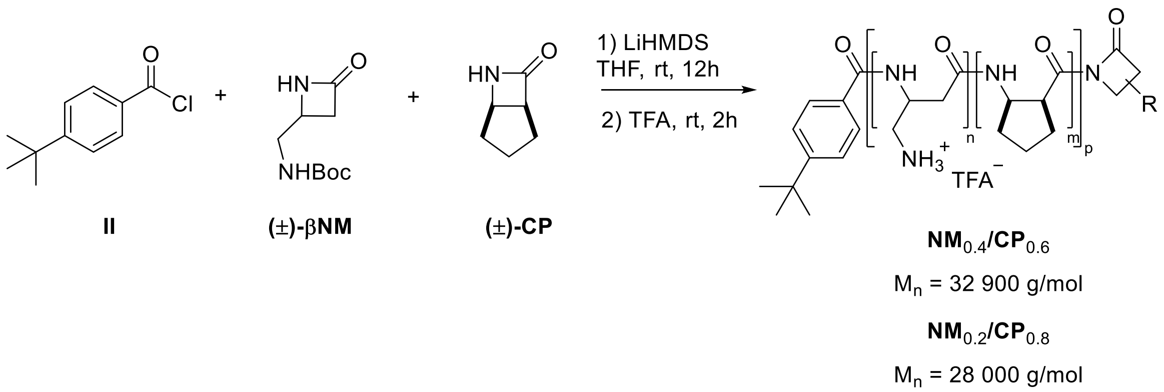


**Figure S2.** Synthesis of TFA-salts of gene delivery Nylon-3 polymers (NM/CP) in presence of a co-initiator II and a base LiHMDS. R = side chain groups of NM or CP. Reproduced with permission from [1]. Copyright American Chemical Society, 2014

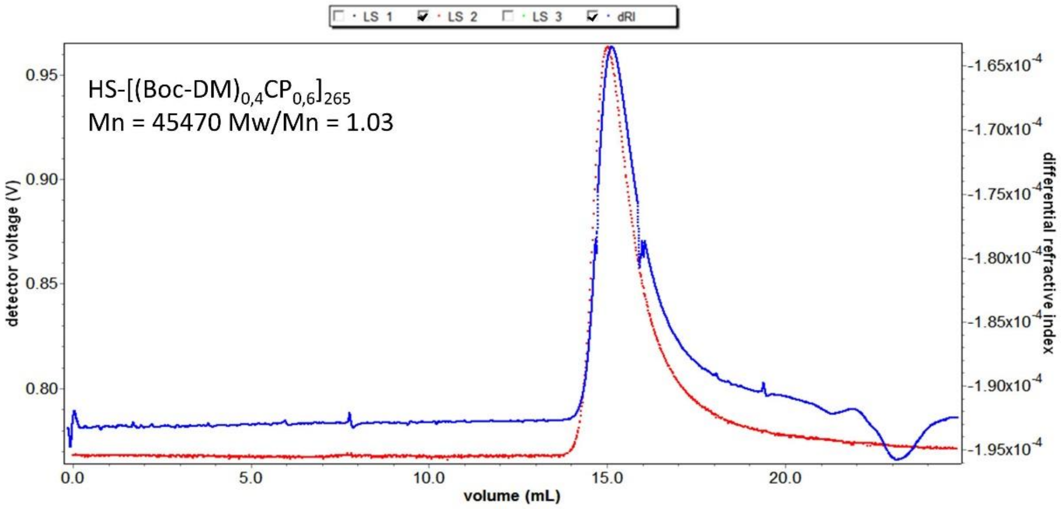


**Figure S3.** GPC chromatograph of Boc-protected HS-[(Boc-DM)$_{0.4}$CP$_{0.6}$]$_{265}$ copolymer measured with light-scattering (red) and refractive index (blue) detectors, mobile phase: THF.

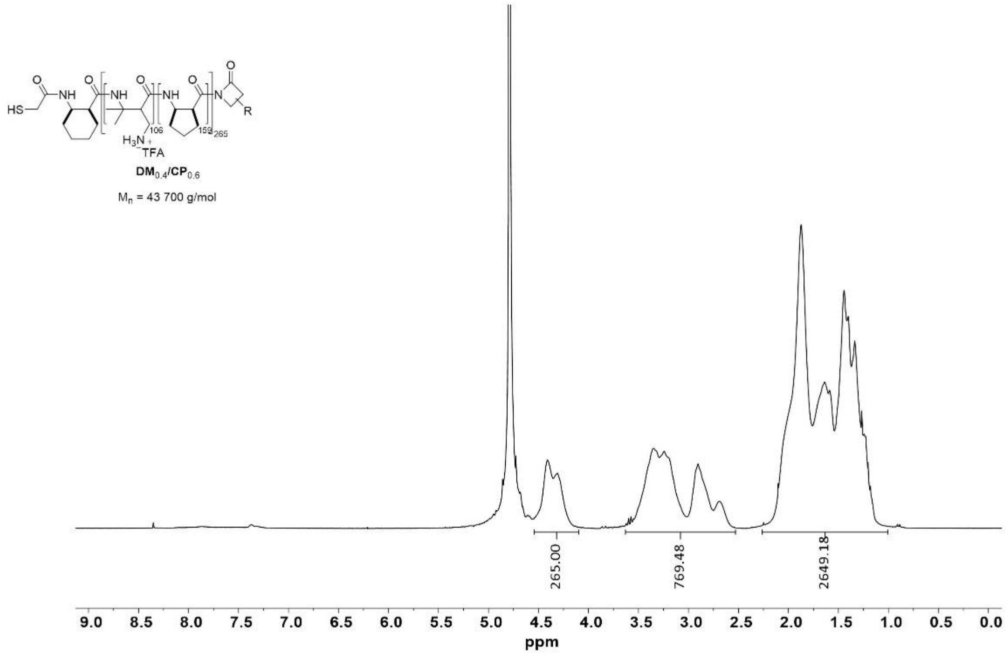


**Figure S4.** $^{1}$H-NMR spectrum of unprotected DM$_{0.4}$/CP$_{0.6}$ polymer measured in $D_2O$ (300 MHz, 512 scans).

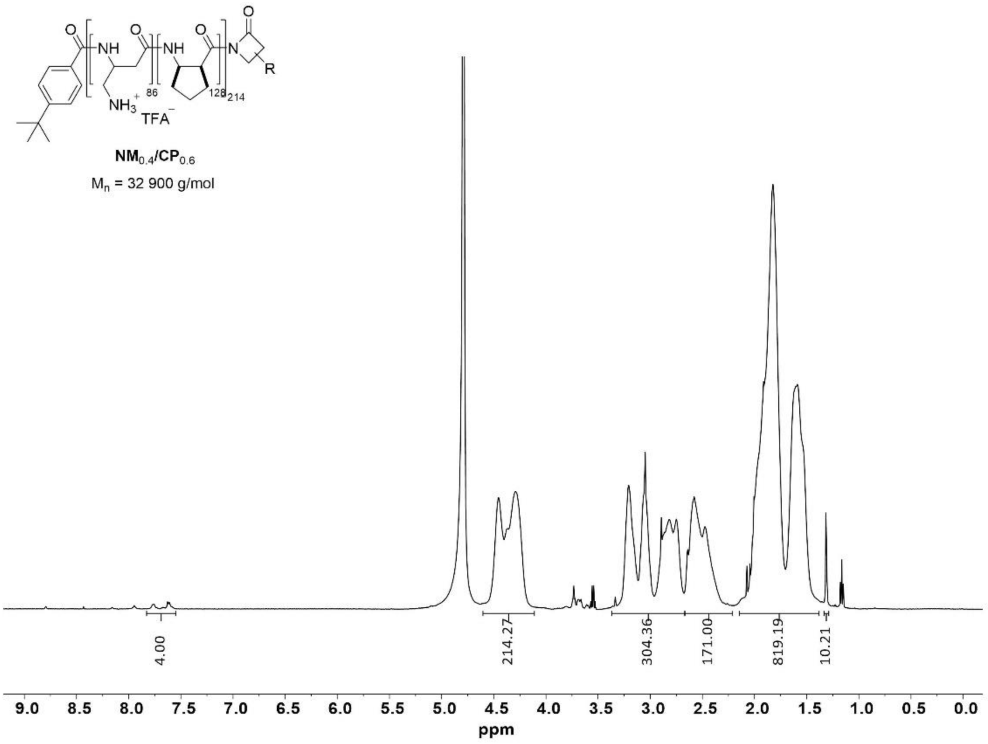


**Figure S5.**$^{1}$H-NMR spectrum in $D_2O$ of unprotected $NM_{0.4}/CP_{0.6}$ polymer measured in $D_2O$ (500 MHz, 126 scans).

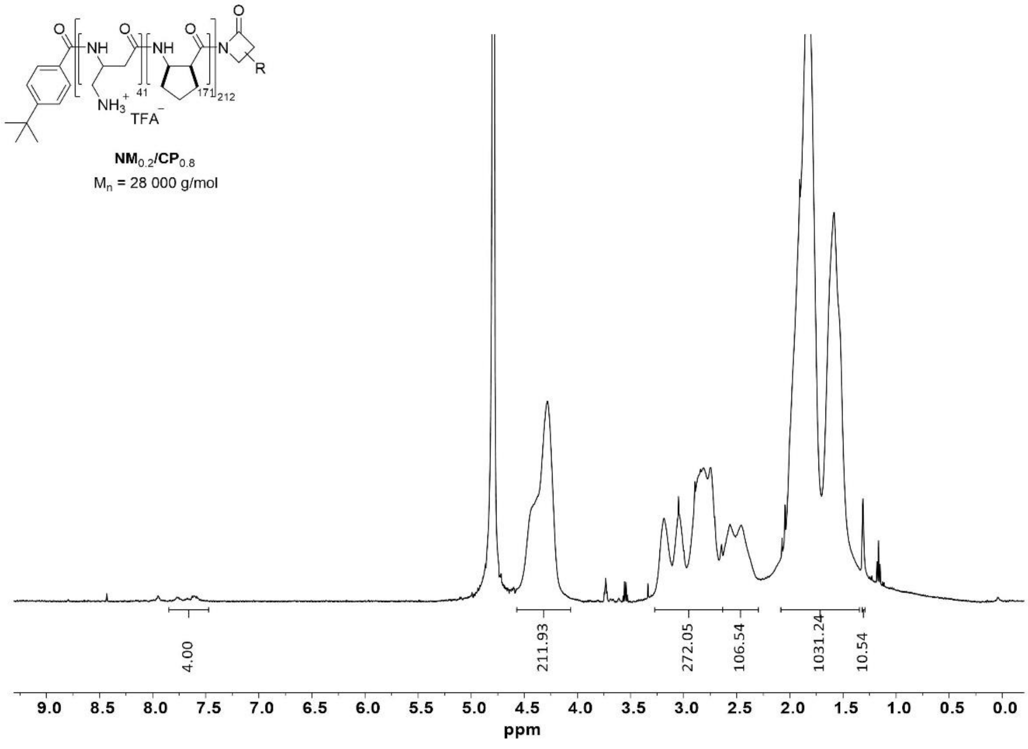


**Figure S6.**$^{1}$H-NMR spectrum of unprotected $NM_{0.2}/CP_{0.8}$ polymer measured in $D_2O$ (500 MHz, 126 scans).

## 2. Polyplex characterization

### 2.1. N/P ratio optimization

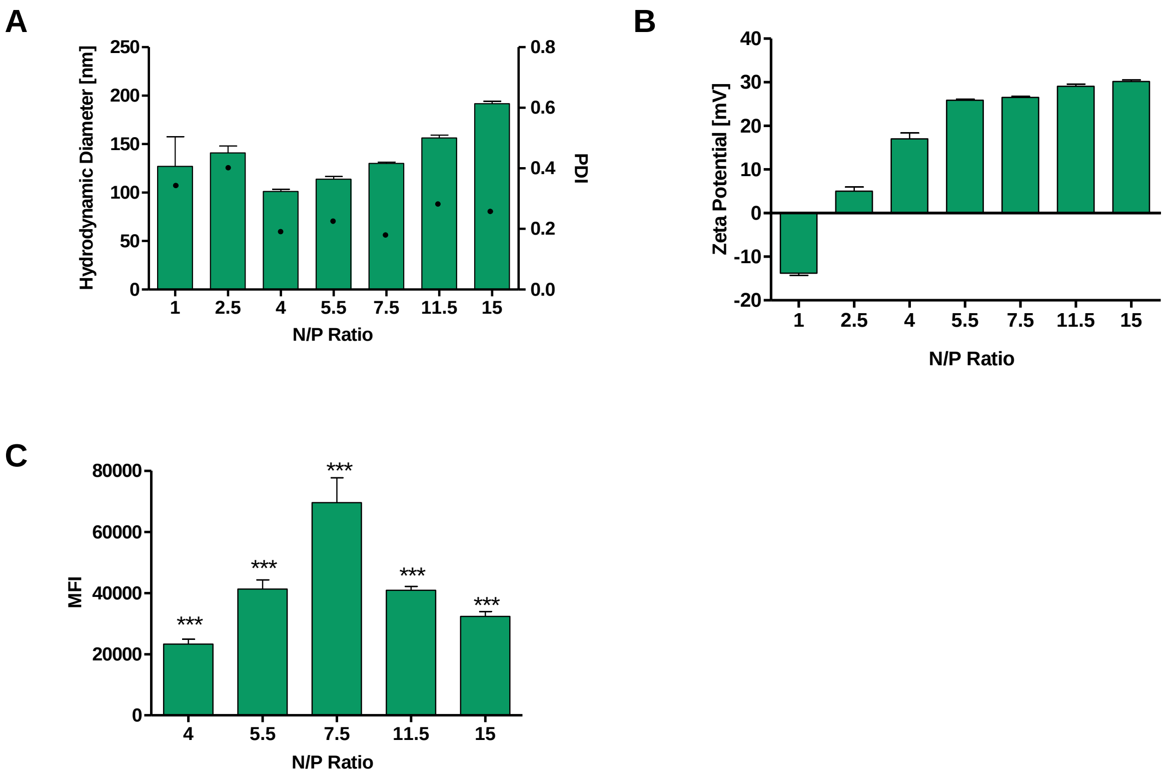


**Figure S7.** (**A**) Hydrodynamic diameters investigated by DLS (left y-axis) and polydispersity indices (PDI, right y-axis), (**B**) zeta potentials measured by LDA and (**C**) MFIs of $NM_{0.2}/CP_{0.8}$ cells treated with respective polyplexes determined by flow cytometry. (Data points indicate mean ± SD, $n$ = 3).

### 2.2. Size measurements by TRPS

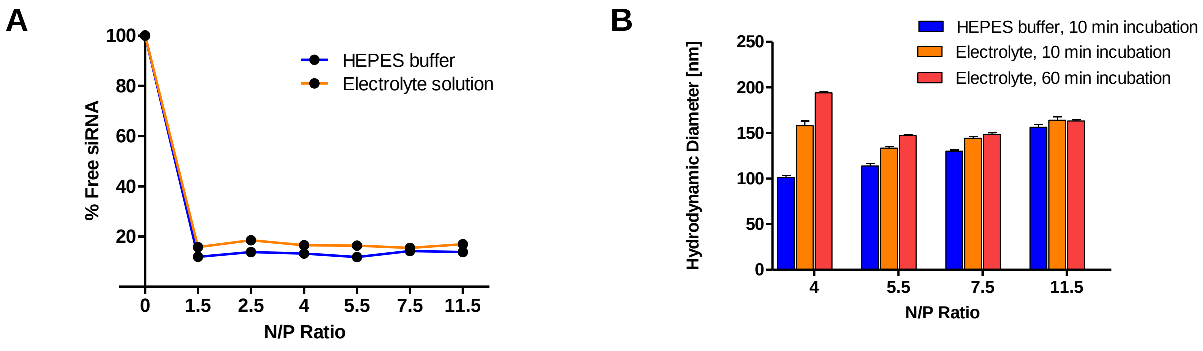


**Figure S8.** (**A**) siRNA encapsulation profiles of $NM_{0.2}/CP_{0.8}$ polyplexes prepared in 10 mM HEPES buffer at N/P 4 and 1X diluted with either 10 mM HEPES buffer or TRPS electrolyte solution (30 mM HEPES, 100 mM potassium chloride, 2 mM EDTA and 0.03% Tween®20). 100% values (N/P = 0) are determined by fluorescence of uncondensed siRNA. (Data points indicate mean, $n$ = 3). (**B**) DLS measurements of $NM_{0.2}/CP_{0.8}$ polyplexes prepared in 10 mM HEPES buffer at N/P 4 and 1X diluted with either 10 mM HEPES buffer or TRPS electrolyte solution, measured after 10 and 60 min incubation period (Data points indicate mean ± SD, $n$ = 3).

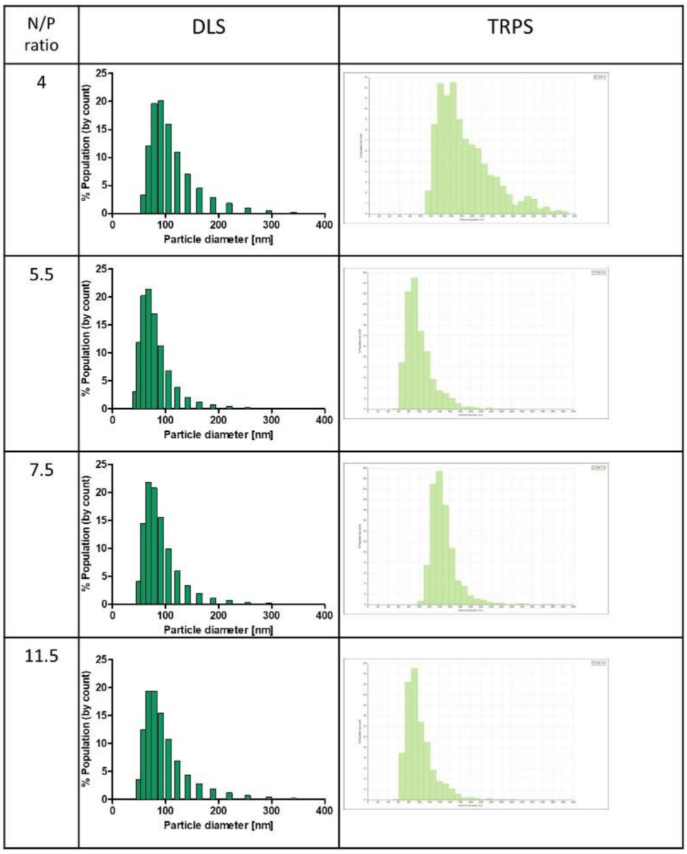


**Figure S9.** Number-weighted size distributions of $NM_{0.2}/CP_{0.8}$ polyplexes at N/P ratios 5, 5.5, 7.5 and 11.5. 1:1 diluted with electrolyte solution and measured by DLS and TRPS, respectively.

## 3. Quantification of Cellular Uptake

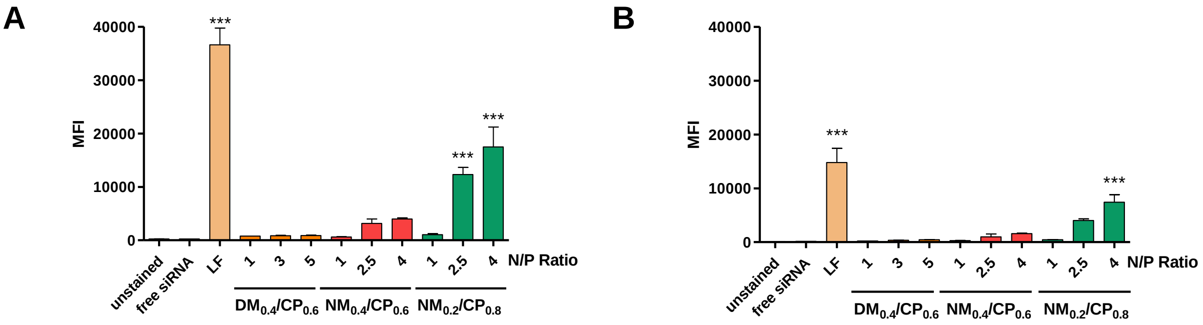


**Figure S10.** Cellular uptake of polyplexes performed at various N/P ratios in (**A**) H1299 cells and (**B**) U87 cells after 5 h incubation as determined by flow cytometry presented as median fluorescence intensity. Negative control: untreated cells and cells treated with free siRNA, positive control: cells transfected with Lipofectamin (LF) lipoplexes. (Data points indicate mean ± SD, *n* = 3, two-way ANOVA with Bonferroni post-hoc test, ***$p < 0.005$).

## 4. Route of cellular Uptake

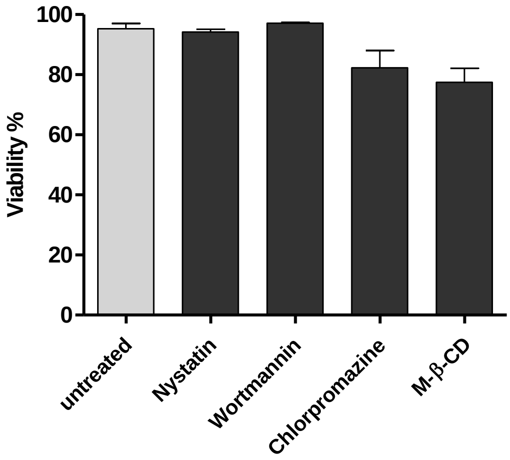


**Figure S11.** U87 cell viabilities after treatment with nystatin (10 µg/mL), wortmannin (12ng/mL), chlorpromazine (10 µg/mL) and methyl-beta-cyclodextrin (3 mg/mL); determined by trypan blue staining. Number of living and dead cells was counted in a Neubauer chamber using an Axio Vert.A1 microscope. The percentage of viable cells was calculated. (Results are given as mean ± SD, $n$ = 3)

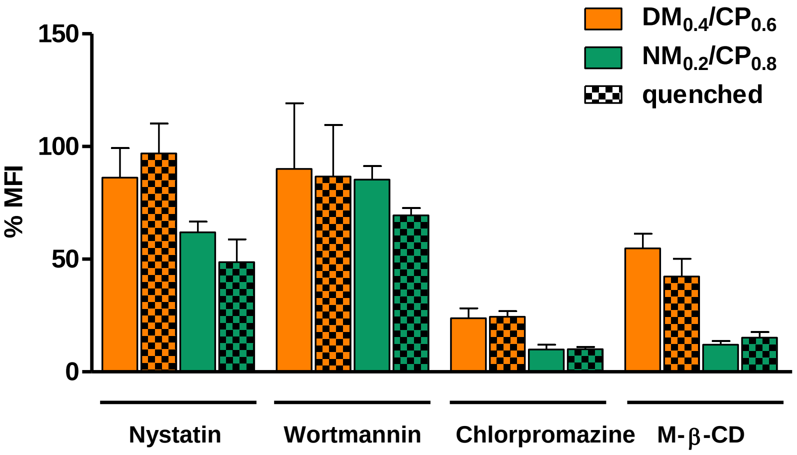


**Figure S12.** Cellular uptake of polyplexes ($DM_{0.4}/CP_{0.6}$ polyplexes: N/P ratio= 5 and $NM_{0.2}/CP_{0.8}$ polyplexes: N/P ratio = 4) after treatment with nystatin (10 µg/ml), wortmannin (12 ng/mL), chlorpromazine (10 µg/mL) and methyl-β-cyclodextrin (M-β-CD) (3 mg/mL) conducted with and without trypan quenching as evaluated by flow cytometry and presented as MFI. (Results are shown as mean ± SD as percentage of median fluorescence intensity related to not inhibited samples, $n$ = 3).

## References

1. Liu, R.; Masters, K.S. Polymer chain length effects on fibroblast attachment on nylon-3-modified surfaces. *Biomacromolecules*, **2012**, *13*, 1100–1105,.
2. Liu, R.; Chen, X. Structure-activity relationships among antifungal nylon-3 polymers: Identification of materials active against drug-resistant strains of Candida albicans. *Journal of the American Chemical Society*, **2014**, *136*, 4333–4342.
